# Serum CXCL10 levels at the start of the second course of atezolizumab plus bevacizumab therapy predict therapeutic efficacy in patients with advanced BCLC stage C hepatocellular carcinoma: A multicenter analysis

**DOI:** 10.1002/cam4.6876

**Published:** 2023-12-22

**Authors:** Takanori Suzuki, Kentaro Matsuura, Yuta Suzuki, Fumihiro Okumura, Yoshihito Nagura, Satoshi Sobue, Sho Matoya, Tomokatsu Miyaki, Yoshihide Kimura, Atsunori Kusakabe, Satoshi Narahara, Takayuki Tokunaga, Katsuya Nagaoka, Keita Kuroyanagi, Hayato Kawamura, Kayoko Kuno, Kei Fujiwara, Shunsuke Nojiri, Hiromi Kataoka, Yasuhito Tanaka

**Affiliations:** ^1^ Department of Gastroenterology and Metabolism Nagoya City University Graduate School of Medical Sciences Nagoya Japan; ^2^ Department of Gastroenterology Gifu Prefectural Tajimi Hospital Gifu Japan; ^3^ Department of Gastroenterology Kasugai Municipal Hospital Kasugai Japan; ^4^ Department of Gastroenterology Toyokawa City Hospital Toyokawa Japan; ^5^ Department of Gastroenterology Nagoya City University West Medical Center Nagoya Japan; ^6^ Department of Gastroenterology Japanese Red Cross Aichi Medical Center Nagoya Daini Hospital Nagoya Japan; ^7^ Department of Gastroenterology and Hepatology, Faculty of Life Sciences Kumamoto University Kumamoto Japan

**Keywords:** atezolizumab, bevacizumab, C‐X‐C motif chemokine ligand 10, cytokine, hepatocellular carcinoma

## Abstract

**Background & Aims:**

Relationships of serum C‐C motif chemokine ligand 5 (CCL5) and C‐X‐C motif chemokine ligand 10 (CXCL10) levels with hot immune features have been reported in patients with hepatocellular carcinoma (HCC). Therefore, we examined the utility of their levels for predicting the efficacy of atezolizumab plus bevacizumab (Atez/Bev) in patients with HCC.

**Design:**

In total, 98 patients with HCC treated with Atez/Bev were enrolled, and their initial responses were evaluated at least once via dynamic computed tomography or magnetic resonance imaging. Serum CCL5 and CXCL10 levels were assessed by enzyme‐linked immunosorbent assay before treatment and at the start of the second course of Atez/Bev therapy, and their relationships with treatment efficacy were determined.

**Results:**

No analyzed factor was associated with the initial therapeutic response. Among the 56 patients with Barcelona Clinic Liver Cancer (BCLC) stage C, serum CXCL10 levels at the beginning of course two (CXCL10‐2c) tended to be higher in responders than in non‐responders in the initial evaluation, and its optimal cutoff level of 690 pg/mL could be used to stratify patients regarding overall survival (OS; high vs. low: not reached vs. 17.6 months, *p* = 0.034) and progression‐free survival (high vs. low: 13.6 vs. 5.1 months, *p* = 0.014). In multivariate analysis, high CXCL10 levels and neutrophil‐to‐lymphocyte ratios at the start of course two and Child–Pugh stage A at baseline were independent predictive factors of improved OS.

**Conclusions:**

Serum CXCL10‐2c levels were predictive of Atez/Bev efficacy in patients with BCLC stage C HCC.

## 1 INTRODUCTION

Hepatocellular carcinoma (HCC) is the sixth most common cancer globally.^1^ Patients with HCC have poor prognoses because of concomitant chronic liver disease, late diagnoses, and frequent recurrence or progression after treatment.^2^, ^3^ To date, a number of systemic chemotherapeutic regimens have been developed for unresectable HCC.^4^, ^5^, ^6^, ^7^, ^8^ The combination of the immune checkpoint inhibitor (ICI) atezolizumab and the anti‐vascular endothelial growth factor drug bevacizumab (Atez/Bev) was authorized in 2020 as a first‐line treatment for HCC.^9^ In the IMbrave150 trial, Atez/Bev therapy displayed greater efficacy than sorafenib in patients with unresectable HCC.^9^, ^10^ However, prognostic biomarkers have not been fully established for patients treated with Atez/Bev for unresectable HCC.

Although ICIs have been approved for use in various cancers, their response rates are low in several cancers including breast cancer, prostate cancer, and HCC.^11^, ^12^, ^13^ In prior research, a T cell‐inflamed tumor microenvironment (TME) was linked to better responses to ICIs, whereas immune‐excluded tumors were prone to ICI resistance.^14^ HCC with a T cell‐inflamed TME comprises approximately 35% of all cases, and the profile of the inflamed class of HCC includes higher expression of C‐C motif chemokine ligand 5 (CCL5), CCL4, and other cytokines involved in lymphocyte chemotaxis, such as C‐X‐C motif chemokine ligand 9 (CXCL9), CXCL10, and CXCL11.^15^ Interestingly, a previous study detected elevated plasma CXCL9 and CXCL10 levels in mice that responded to ICI therapy, and similar results were recorded in patients with melanoma.^16^ In addition, Hosoda et al. revealed that low serum CXCL9 levels at baseline predicted early disease progression in patients treated with Atez/Bev for unresectable HCC.^17^ These results suggest that the plasma levels of CXCR3 ligands, such as CXCL9 and CXCL10, could be early biomarkers of ICI responsiveness. Furthermore, recent studies identified an association of high circulating interleukin‐6 levels with poor clinical outcomes and impaired T‐cell function in patients treated with Atez/Bev for unresectable HCC.^18^, ^19^

In this background, we hypothesized that the levels of cytokines/chemokines related to the inflamed class of HCC could be altered by therapy and that they better reflect the immunological status of patients, highlighting their potential utility as predictors of ICI effectiveness. Among various cytokines and chemokines, serum CCL5 and CXCL10 have been linked to hot immune features, and patients with advanced HCC and high CCL5 and CXCL10 levels could experience greater benefit from ICI therapy.^15^ Therefore, we retrospectively investigated whether serum CCL5 and CXCL10 levels could predict Atez/Bev efficacy in patients with advanced HCC.^15^

## 2 METHODS

### 2.1 Patients and study design

This multicenter, retrospective, observational study included 98 patients with HCC. All patients received Atez/Bev therapy irrespective of the line of systemic chemotherapy, and their initial responses were evaluated by dynamic computed tomography (CT) or magnetic resonance imaging (MRI) at least once between October 2020 and January 2023 at seven Japanese institutions (Nagoya City University Hospital [*n* = 20], Gifu Prefectural Tajimi Hospital [*n* = 8], Toyokawa City Hospital [*n* = 6], Kasugai Municipal Hospital [*n* = 5], Nagoya City University West Medical Center [*n* = 5], Japanese Red Cross Aichi Medical Center Nagoya Daini Hospital [*n* = 2], Kumamoto University Hospital [*n* = 52]). Serum samples were obtained at baseline and the beginning of the second course of Atez/Bev therapy and stored at −80°C.

### 2.2 Diagnosis and treatment of HCC

HCC was diagnosed on the basis of increases in α‐fetoprotein levels and the results of dynamic CT, MRI, and/or pathology. Patients positive for hepatitis B virus (HBV) surface antigen or hepatitis C virus (HCV) antibody and those with a history of alcohol abuse (≥60 g/day) were considered to have HCC attributable to HBV, HCV, and alcohol, respectively. The Barcelona Clinic Liver Cancer (BCLC) criteria were used for HCC staging.^20^

The treatment regimen consisted of 1200 mg of atezolizumab and 15 mg/kg body weight bevacizumab every 3 weeks.^9^ Atez/Bev therapy was discontinued following unacceptable or serious adverse events or clinical tumor progression. In certain BCLC stage A patients, Atez/Bev therapy was initiated when cardiopulmonary function prevented hepatic resection, percutaneous ablation therapy was difficult because of intrahepatic vascular effects or organs near the liver, or transarterial chemoembolization (TACE) was difficult in patients allergic to iodinated contrast agent or those with an HCC type unsuitable for TACE.

### 2.3 Laboratory tests and evaluation of liver function

Hematologic and blood chemistry tests were performed using standard assays. The neutrophil‐to‐lymphocyte ratio (NLR) was calculated using the absolute neutrophil and lymphocyte counts in peripheral blood. We assessed liver function using the Child–Pugh classification system and albumin–bilirubin (ALBI) score, which was calculated using serum albumin and total bilirubin levels based on the following formula: ALBI score = (log10 bilirubin [μmol/L] × 0.66) + (albumin [g/L] × −0.085).^21^

### 2.4 Ethical standards

Each patient provided written informed consent prior to enrollment. The study protocol was approved by the Institutional Review Boards of Nagoya City University (acceptance number: 60‐21‐0065) and Kumamoto University (acceptance number: 2265) and implemented according to the Declaration of Helsinki.

### 2.5 Chemokine assays

Serum CCL5 and CXCL10 levels were measured using commercial enzyme‐linked immunosorbent assay kits per the manufacturer's protocol (R&D Systems, Minneapolis, MN, USA).

### 2.6 Assessment of responses to therapy

Therapeutic response was determined using the Response Evaluation Criteria in Solid Tumors (RECIST) ver. 1.1.^22^ Therapeutic response was initially assessed using dynamic CT or Gd‐EOB‐DTPA‐MRI (EOB‐MRI) approximately 6–9 weeks after treatment initiation and repeated every 6–9 weeks in treatment responders. The objective response rate (ORR) was defined as the sum of the complete response (CR) and partial response (PR) rates, and the disease control rate (DCR) was defined as the sum of the CR, PR, and stable disease (SD) rates. Progression‐free survival (PFS) was defined as the time from the start of Atez/Bev therapy to the date of documented progression per RECIST v. 1.1 or death from any cause. If the patient received another systemic chemotherapy or he/she was lost to follow‐up before progressive disease (PD) was documented, PFS was censored at the date of the last observation. Overall survival (OS) was calculated as the time from the start of Atez/Bev therapy to death or the last follow‐up.

### 2.7 Statistical analysis

Categorical variables were compared between the groups using Fisher's exact test, and non‐categorical variables were analyzed using the Mann–Whitney *U‐test*. Receiver operating characteristic (ROC) curve analysis was conducted, and the area under the curve (AUC) was calculated to identify the optimal serum CXCL10 levels for discriminating responders (CR + PR) and non‐responders (SD + PD) using the Youden index (Youden index = sensitivity + specificity – 1; range = 0–1). Cumulative OS and PFS were analyzed using the Kaplan–Meier method, and differences were assessed using the log‐rank test. Multivariate analysis was performed using the stepwise Cox proportional hazard model to identify factors associated with PFS and OS. Correlation coefficients were calculated using Pearson's correlation test. All reported *p*‐values were two‐sided, and *p* < 0.05 denoted significance. Statistical analysis was performed using EZR (Easy R, Saitama Medical Center, Jichi Medical University, Saitama, Japan), a modified version of R commander (version 1.61).^23^

## 3 RESULTS

### 3.1 Patient characteristics

As presented in Table 1, the cohort included 78 men (80%) and 20 women (20%) with a median age of 73 years. The BCLC stage was A, B, and C in 6 (6%), 36 (37%), and 56 patients (57%), respectively. Meanwhile, 72 (73%), 19 (19%), 4 (4%), 2 (2%), and 1 (1%) patient received first‐, second‐, third‐, fourth‐, and sixth‐line systemic chemotherapy, respectively. The median ALBI score was −2.35. The details of the regimens provided before and after Atez/Bev therapy are presented in Figure S1. The median baseline serum CXCL10 level (CXCL10‐pre) was lower than that at the start of the second course (CXCL10‐2c; 217.5 pg/mL vs. 357.0 pg/mL). Similarly, the median baseline serum CCL5 level (CCL5‐pre) was lower than that at the start of the second course (CCL5‐2c; (20,250 pg/mL vs. 27,950 pg/mL). CXCL10‐pre, CXCL10‐2c, CCL5‐pre, and CCL5‐2c levels did not significantly differ by etiology (viral vs. non‐viral; Figures S2 and S3) or treatment line (Figures S4 and S5).

**TABLE 1:** Clinical characteristics of the study patients.

Characteristics	*n* = 98
Baseline	
Age, years	73 (66–78)
Gender, male/female	78 (80%)/20 (20%)
BMI, kg/m^2^	23.4 (20.8–26.8)
ECOG PS, 0/1	91 (93%)/7 (7%)
Etiology, HBV/HCV/alcohol/others	18 (18%)/25 (26%)/16 (16%)/39 (40%)
Treatment line, 1st/2nd/3rd/4th/6th	72 (73%)/19 (19%)/4 (4%)/2 (2%)/1 (1%)
WBCs, μL	5000 (3800–6300)
Neutrophils, μL	3170 (2092–4000)
Lymphocytes, μL	1234 (896–1530)
NLR	2.56 (1.61–3.84)
Hb, g/dL	12.7 (11.5–14.2)
PLTs, × 10^4^/μL	15.2 (11.1–19.2)
PT, %	98.1 (87.1–106.8)
AST, U/L	39 (27–51)
ALT, U/L	27 (19–39)
Alb, g/dL	3.7 (3.4–4.0)
T.Bil, mg/dL	0.8 (0.6–1.2)
AFP, ng/mL	15.8 (4.9–310.9)
PIVKA‐II, mAU/mL	248.0 (49.0–1732.5)
ALBI score	−2.35 (−2.65 to −2.09)
mALBI grade, 1/2a/2b/3	29 (30%)/31 (32%)/36 (37%)/2 (2%)
Child–Pugh score, 5/6/7/8	49 (50%)/35 (36%)/12 (12%)/2 (2%)
BCLC stage, A/B/C	6 (6%)/36 (37%)/56 (57%)
MVI, yes/no	26 (27%)/72 (73%)
Extrahepatic metastasis, yes/no	45 (46%)/53 (54%)
CXCL10, pg/mL	217.5 (163.5–330.8)
CCL5, pg/mL	20,250 (14,350–28,300)
Start of the second course	
Neutrophils, μL	2862 (1979–3938)
Lymphocytes, μL	1200 (864–1.698)
NLR	2.51 (1.54–3.79)
CXCL10, pg/mL	357.0 (232.5–699.0)
CCL5, pg/mL	27,950 (15,400–37,125)

*Note*: Data from all patients are expressed as numbers for categorical data and medians (first–third quartiles) for non‐categorical data.

Abbreviations: AFP, α‐fetoprotein; Alb, albumin; ALBI score, albumin–bilirubin score; ALT, alanine transaminase; AST, aspartate transaminase; BCLC, Barcelona Clinic Liver Cancer; BMI, body mass index; CCL5, C‐C motif chemokine ligand 5; CXCL10, C‐X‐C motif chemokine ligand 10; ECOG PS, ECOG performance status; Hb, hemoglobin; HBV, hepatitis B virus; HCV, hepatitis C virus; mALBI grade, modified albumin–bilirubin grade; MVI, major vascular invasion; NLR, neutrophil‐to‐lymphocyte ratio; PIVKA‐II, protein induced by vitamin K absence or antagonist‐II; PLTs, platelets; PT, prothrombin time; T.Bil, total bilirubin; WBCs, white blood cells.

Of the 98 patients with HCC, 1 (1%), 21 (21%), 61 (62%), and 15 (15%) had CR, PR, SD, and PD, respectively, in the initial evaluation. Therefore, the ORR and DCR at the initial evaluation were 22% and 85%, respectively. The median follow‐up period of this study was 13.1 months (interquartile range: 7.6–18.6), and median PFS and OS were 8.3 and 21.4 months, respectively (Figure S6).

### 3.2 Patient characteristics stratified by the initial therapeutic response

The characteristics of all patients stratified by the initial therapeutic response are summarized in Table S1. No factors, including serum CXCL10‐pre, CXCL10‐2c, CCL5‐pre, and CCL5‐2c levels, significantly differed between responders and non‐responders.

**FIGURE 1:**
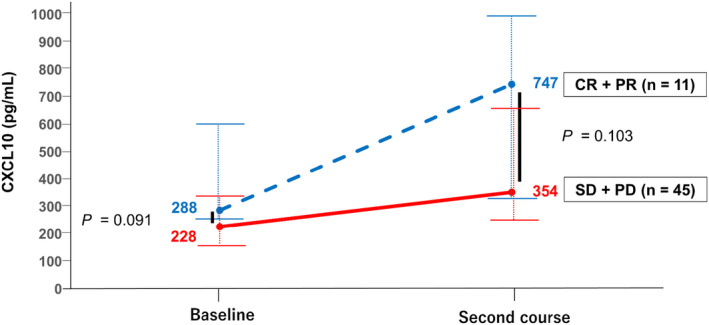
Changes of serum CXCL10 levels from baseline to the start of the second course stratified by the initial therapeutic response in patients with BCLC stage C HCC. Changes of serum CXCL10 levels in responders (CR + PR, *n* = 11) and non‐responders (SD + PD, *n* = 45) from baseline to the start of the second course of Atez/Bev therapy. The dotted lines represent the median serum CXCL10 level of responders with BCLC stage C HCC, whereas solid lines represent the median serum CXCL10 levels of non‐responders with BCLC stage C HCC. The horizontal lines represent the interquartile range of the data. *p*‐values were calculated using the Mann–Whitney *U*‐test. Atez/Bev, atezolizumab plus bevacizumab; BCLC, Barcelona Clinic Liver Cancer; CR, complete response; CXCL10, C‐X‐C motif chemokine ligand 10; HCC, hepatocellular carcinoma; PD, progressive disease; PR, partial response; SD, stable disease.

Next, we compared serum CCL5 and CXCL10 levels by BCLC stage. CXCL10‐pre levels did not differ between BCLC stages A/B and BCLC stage C (192 pg/mL vs. 236 pg/mL, *p* = 0.278), whereas serum CXCL10‐2c levels were numerically higher in patients with BCLC stage C than in those with BCLC stages A/B (308 pg/mL vs. 380 pg/mL, *p* = 0.143; Figure S7). CCL5‐pre levels did not differ between BCLC stages A/B and BCLC stage C (19,950 pg/mL vs. 20,450 pg/mL, *p* = 0.331). Meanwhile, serum CCL5‐2c levels were higher in patients with BCLC stage C than in those with BCLC stages A/B (21,650 pg/mL vs. 30,500 pg/mL, *p* = 0.041; Figure S8). Therefore, we focused our analyses on the associations of serum CXCL10‐2c and CCL5‐2c levels with treatment efficacy in patients with BCLC stage C HCC. The clinical characteristics of the 56 patients with BCLC stage C HCC and their characteristics stratified by the initial therapeutic response are summarized in Table S2 and Table 2. As presented in Table 2, no factors at baseline or at the start of the second course, including serum CCL5‐pre and CCL5‐2c levels, were associated with the initial therapeutic response (Figure S9), but serum CXCL10‐pre (288 pg/mL vs. 228 pg/mL, *p* = 0.091) and CXCL10‐2c levels (CXCL10‐2c: 747 pg/mL vs. 354 pg/mL, *p* = 0.103) were higher in responders than in non‐responders. Moreover, serum CXCL10 levels greatly increased from baseline to the start of the second course in responders compared with the findings in non‐responders (Figure 1).

**TABLE 2:** Comparison of the characteristics of responders and non‐responders at the initial evaluation among patients with Barcelona Clinic Liver Cancer stage C hepatocellular carcinoma.

Characteristics	CR + PR (*n* = 11)	SD + PD (*n* = 45)	*p*‐value
Baseline			
Age, years	78 (71–81)	71 (64–76)	0.137
Gender, male/female	9 (82%)/2 (18%)	36 (80%)/9 (20%)	1.000
Etiology, viral/non‐viral	4 (36%)/7 (64%)	20 (44%)/25 (56%)	0.741
Line of systemic chemotherapy, 1st/≥2nd	8 (73%)/3 (27%)	29 (64%)/16 (36%)	0.732
BMI, kg/m^2^	24.5 (22.4–26.4)	22.8 (20.5–24.6)	0.201
WBCs, /μL	5400 (4900–6650)	5300 (4000–6800)	0.550
Neutrophils, /μL	3626 (3165–4356)	3300 (2305–4300)	0.439
Lymphocytes, /μL	1100 (728–1503)	1278 (806–1634)	0.599
NLR	3.18 (2.18–4.88)	2.61 (1.61–4.37)	0.515
PT, %	98.0 (89.4–105.1)	101.0 (87.0–108.9)	0.773
AST, U/L	39 (35–55)	40 (27–51)	0.522
ALT, U/L	25 (18–42)	27 (18–37)	0.828
Alb, g/dL	3.7 (3.3–3.9)	3.5 (3.4–3.9)	0.942
T.Bil, mg/dL	0.8 (0.7–1.0)	0.8 (0.7–1.2)	0.664
AFP, ng/mL	833.3 (13.0–3029.4)	18.4 (4.7–2280.0)	0.205
PIVKA‐II, mAU/mL	218 (30–8397)	923 (60–5653)	0.536
ALBI score	−2.28 (−2.57 to − 2.09)	−2.34 (−2.56 to − 2.09)	0.877
mALBI grade, 1/2a/2b/3	3 (27%)/3 (27%)/5 (45%)/0 (0%)	11 (24%)/13 (29%)/20 (44%)/1 (2%)	1.000
Child–Pugh score: 5/6/7/8	6 (55%)/4 (36%)/1 (9%)/0 (0%)	20 (44%)/16 (36%)/8 (18%)/1 (2%)	0.923
Extrahepatic metastasis, yes/no	8 (73%)/3 (27%)	37 (82%)/8 (18%)	0.673
CXCL10, pg/mL	288.0 (232.5–603.0)	228.0 (171.0–324.0)	0.091
CCL5, pg/mL	16,500 (15,050–27,100)	21,900 (13,900–34,900)	0.503
Start of the second course			
Neutrophils, /μL	3154 (2598–3591)	3226 (2147–4666)	0.636
Lymphocytes, /μL	1580 (1161–1978)	1121 (695–1663)	0.185
NLR	1.79 (1.37–3.16)	2.88 (1.95–4.65)	0.158
CXCL10, pg/mL	747.0 (366.0–997.5)	345.0 (252.0–669.0)	0.103
CCL5, pg/mL	27,900 (20,550–36,350)	31,700 (19,300–45,600)	0.726

*Note*: Data from all patients are expressed as numbers for categorical data and medians (first–third quartiles) for non‐categorical data. Categorical variables were compared between the groups using Fisher's exact test, and non‐categorical variables were compared using the Mann–Whitney *U‐test*.

Abbreviations: AFP, α‐fetoprotein; Alb, albumin; ALBI score, albumin–bilirubin score; ALT, alanine transaminase; AST, aspartate transaminase; BMI, body mass index; CCL5, C‐C motif chemokine ligand 5; CR, complete response; CXCL10, C‐X‐C motif chemokine ligand 10; mALBI grade, modified albumin–bilirubin grade; NLR, neutrophil‐to‐lymphocyte ratio; PD, progressive disease; PIVKA‐II, protein induced by vitamin K absence or antagonist‐II; PR, partial response; PT, prothrombin time; SD, stable disease; T.Bil, total bilirubin; WBCs, white blood cells.

* *p* < 0.05.

** *p* < 0.005.

### 3.3 Appropriate serum CXCL10 for predicting therapeutic response in patients with BCLC stage C HCC

ROC curve analyses were performed to discriminate responders from non‐responders at the time of the initial therapeutic response evaluation, and the diagnostic utility of serum CXCL10‐pre and CXCL10‐2c levels were compared. The AUCs of serum CXCL10‐pre and CXCL10‐2c levels were 0.667 and 0.661, respectively (Figure S10), and their optimal cutoffs were 231 (positive predictive value [PPV] = 29%, negative predictive value [NPV] = 92%, sensitivity = 0.511, specificity = 0.818) and 690 pg/mL (PPV = 39%, NPV = 89%, sensitivity = 0.756, specificity = 0.636), respectively. When we categorized the patients with BCLC stage C HCC into two groups based on serum CXCL10‐2c levels, the CXCL10‐2c high group (≥690 pg/mL) included a higher proportion of initial responders than the CXCL10‐2c low group (<690 pg/mL, *p* = 0.027; Table 3).

**TABLE 3:** Comparison of the characteristics of patients with Barcelona Clinic Liver Cancer stage C hepatocellular carcinoma stratified by serum CXCL10 levels at the start of the second course.

Characteristics	CXCL10‐2c high (≥690 pg/mL) (*n* = 18)	CXCL10‐2c low (<690 pg/mL) (*n* = 38)	*p*‐value
Baseline			
Age, years	75 (71–81)	71 (63–77)	0.114
Gender, male/female	14 (78%)/4 (22%)	31 (82%)/7 (18%)	0.732
Etiology, viral/non‐viral	7 (39%)/11 (61%)	17 (45%)/21 (55%)	0.777
Lines of systemic chemotherapy, 1st/≥2nd	14 (78%)/4 (22%)	23 (61%)/15 (39%)	0.241
BMI, kg/m^2^	23.3 (21.1–24.6)	22.8 (20.4–26.1)	0.965
WBCs, /μL	5500 (4475–6600)	5100 (4025–6875)	0.629
Neutrophils, /μL	3886 (2961–4609)	3120 (2358–4214)	0.330
Lymphocytes, /μL	1242 (701–1564)	1261 (824–1750)	0.505
NLR	3.40 (2.12–5.03)	2.54 (1.54–4.36)	0.342
PT, %	96.3 (87.0–106.6)	102.0 (93.6–110.5)	0.330
AST, U/L	50 (34–72)	38 (27–46)	0.082
ALT, U/L	32 (20–45)	25 (18–34)	0.240
Alb, g/dL	3.5 (3.3–3.7)	3.7 (3.4–3.9)	0.221
T.Bil, mg/dL	0.9 (0.7–1.0)	0.8 (0.6–1.2)	0.731
AFP, ng/mL	46.3 (5.4–5961.5)	52.6 (5.3–2425.4)	0.847
PIVKA‐II, mAU/mL	2204 (71–11,314)	594 (55–2506)	0.494
ALBI score	−2.27 (−2.40 to − 1.94)	−2.34 (−2.61 to − 2.12)	0.285
mALBI grade, 1/2a/2b/3	3 (17%)/6 (33%)/8 (44%)/1 (6%)	11 (29%)/17 (45%)/10 (26%)/0 (0%)	0.432
Child–Pugh score: 5/6/7/8	7 (39%)/6 (33%)/5 (28%)/0 (0%)	19 (50%)/14 (37%)/4 (11%)/1 (3%)	0.409
Extrahepatic metastasis, yes/no	15 (83%)/3 (17%)	30 (79%)/8 (21%)	1.000
CXCL10, pg/mL	477.0 (261.0–741.8)	187.5 (135.0–279.8)	<0.001**
CCL5, pg/mL	18,450 (14,475–33,075)	21,200 (14,400–33,000)	0.888
Initial treatment response: CR + PR/SD + PD	11 (61%)/7 (39%)	34 (89%)/4 (11%)	0.027*
Start of the second course			
Neutrophils, /μL	3012 (2047–3687)	3312 (2496–4631)	0.240
Lymphocytes, /μL	1252 (735–1681)	1168 (738–1700)	0.837
NLR	2.40 (1.61–4.38)	2.82 (1.83–4.46)	0.588
CXCL10, pg/mL	969.0 (816.0–1395.0)	276.0 (214.5–384.8)	<0.001**
CCL5, pg/mL	30,500 (17,675–35,250)	30,350 (19,325–44,125)	0.799

*Note*: Data from all patients are expressed as numbers for categorical data and medians (first–third quartiles) for non‐categorical data. Categorical variables were compared between the groups using Fisher's exact test, and non‐categorical variables were compared using the Mann–Whitney *U‐test*.

Abbreviations: AFP, α‐fetoprotein; Alb, albumin; ALBI score, albumin–bilirubin score; ALT, alanine transaminase; AST, aspartate transaminase; BMI, body mass index; CCL5, C‐C motif chemokine ligand 5; CR, complete response; CXCL10, C‐X‐C motif chemokine ligand 10; mALBI grade, modified albumin–bilirubin grade; NLR, neutrophil‐to‐lymphocyte ratio; PD, progressive disease; PIVKA‐II, protein induced by vitamin K absence or antagonist‐II; PR, partial response; PT, prothrombin time; SD, stable disease; T.Bil, total bilirubin; WBCs, white blood cells.

* *p* < 0.05.

** *p* < 0.005.

### 3.4 Correlations of serum CCL5 and CXCL10 levels with clinical parameters

The correlations of serum CCL5 and CXCL10 levels with various clinical parameters were examined in the entire cohort. As presented in Table S3, serum CCL5‐pre levels were significantly correlated with white blood cells (*r* = 0.368, *p* < 0.001) and neutrophil counts (*r* = 0.415, *p* < 0.001), NLR (*r* = 0.317, *p* < 0.001), and α‐fetoprotein (r = 0.328, *p* < 0.001) and protein induced by vitamin K absence or antagonist‐II levels at baseline (*r* = 0.500, *p* < 0.001). Meanwhile, serum CCL5‐2c levels were significantly correlated with white blood cells (*r* = 0.346, *p* < 0.001), neutrophil (*r* = 0.310, *p* = 0.002), and lymphocyte counts (*r* = 0.254, *p* = 0.013). Serum CXCL10‐pre levels were negatively correlated with the lymphocyte count (*r* = −0.203, *p* = 0.045) and positively correlated with baseline NLR (*r* = 0.248, *p* = 0.014), whereas serum CXCL10‐2c levels were not correlated with any parameters (Table S4).

### 3.5 OS and PFS stratified by serum CXCL10 levels at baseline and at the start of the second course of Atez/Bev therapy in patients with BCLC stage C HCC

**FIGURE 2:**
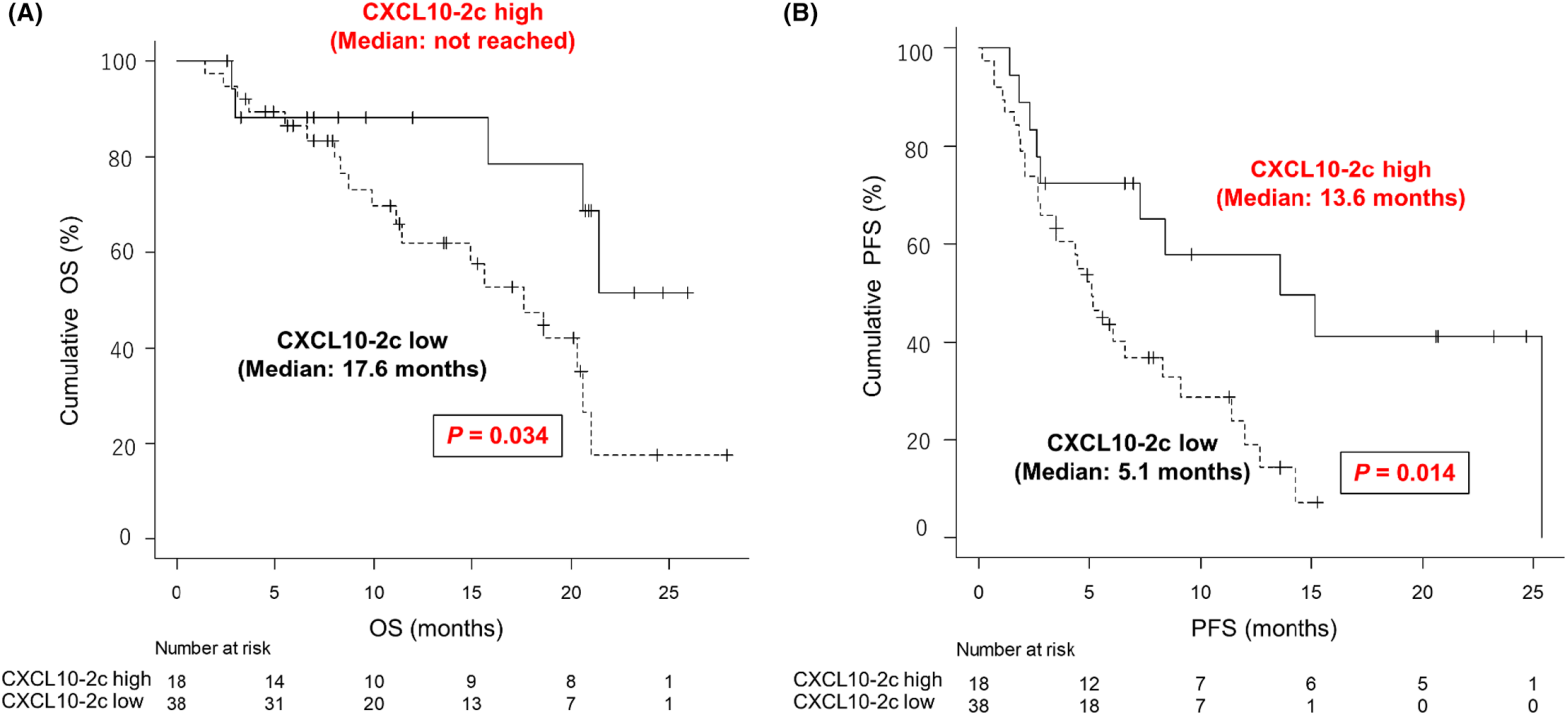
OS and PFS stratified by serum CXCL10 levels at the start of the second course of Atez/Bev therapy. (A) OS stratified by serum CXCL10 levels at the start of the second course. The dotted line represents OS among patients with serum CXCL10‐2c levels <690 pg/mL. The solid line represents OS among patients with CXCL10‐2c levels ≥690 pg/mL. (B) PFS stratified by serum CXCL10 levels at the start of the second course. The dotted line represents PFS among patients with serum CXCL10‐2c levels <690 pg/mL. The solid line represents PFS among patients with serum CXCL10‐2c levels ≥690 pg/mL. *p*‐values were calculated using the log‐rank test. Atez/Bev, atezolizumab plus bevacizumab; CXCL10, C‐X‐C motif chemokine ligand 10; CXCL10‐2c, C‐X‐C motif chemokine ligand 10 at the start of the second course; OS, overall survival; PFS, progression‐free survival.

Figure S11 presents the OS and PFS curves of patients with BCLC stage C HCC stratified by the serum CXCL10‐pre (231 pg/mL) and CXCL10‐2c cutoffs (690 pg/mL). Serum CXCL10‐pre levels were not predictive of median OS (≥231 pg/mL vs. <231 pg/mL: 20.3 months vs. 20.6 months, *p* = 0.550) or PFS (9.1 months vs. 6.1 months, *p* = 0.613). However, serum CXCL10‐2c levels were predictive of median OS (≥690 pg/mL vs. <690 pg/mL, not reached vs. 17.6 months, *p* = 0.034) and PFS (13.6 months vs. 5.1 months, *p* = 0.014; Figure 2). Of the 56 patients with BCLC stage C, 45 had distant metastasis, and serum CXCL10‐2c levels tended to predict median OS (≥690 pg/mL vs. <690 pg/mL, 21.4 vs. 17.6 months, *p* = 0.130) and PFS (13.6 months vs. 5.1 months, *p* = 0.059; Figure S12).

### 3.6 Factors associated with PFS and OS

To identify the factors influencing PFS and OS, we conducted univariate and multivariate analyses. Cutoffs for parameters other than serum CXCL10 levels were reported previously.^24^ Univariate analysis revealed that only serum CXCL10‐2c levels were significantly associated with PFS (≥690 pg/mL vs. <690 pg/mL, hazard ratio [HR] = 0.39 *p* = 0.017; Table S5). Concerning OS, univariate analyses illustrated that NLR, the Child–Pugh score at baseline, and NLR and serum CXCL10 levels at the start of the second course were significantly associated with OS. Considering confounding factors and the results of univariate analysis, multivariate analysis was performed. The Child–Pugh score at baseline (≥6 vs. 5, HR = 2.73; *p* = 0.049), NLR at the start of the second course (≥3 vs. <3, HR = 3.26; *p* = 0.015), and serum CXCL10‐2c levels (≥690 pg/mL vs. <690 pg/mL, HR 0.23; *p* = 0.009) were significantly associated with OS (Table 4).

**TABLE 4:** Factors associated with OS in patients with Barcelona Clinic Liver Cancer stage C hepatocellular carcinoma who received atezolizumab plus bevacizumab.

Factor	Univariate analysis SD (*n* = 62)		Multivariate analysis	
	HR (95% CI)	*p*	HR (95% CI)	*p*‐value
Baseline				
Age, years				
≥70	0.54 (0.24–1.21)	0.131		
<70	1			
Gender				
Male	1.35 (0.45–4.01)	0.589		
Female	1			
Etiology		0.634		
Non‐viral	0.82 (0.37–1.84)			
Viral	1			
Line of systemic chemotherapy				
≥2nd	1.82 (0.81–4.09)	0.145		
1st	1			
NLR				
≥3	2.87 (1.24–6.64)	0.013*		
<3	1			
AFP, ng/mL		0.591		
≥400	1.26 (0.55–2.88)			
<400	1			
Child–Pugh score				
≥6	3.13 (1.30–8.48)	0.012*	2.73 (1.01–7.43)	0.049*
5	1		1	
CXCL10				
≥231	1.28 (0.56–2.93)	0.563		
<231	1			
Start of the second course				
NLR				
≥3	3.37 (1.46–7.79)	0.004**	3.26 (1.26–8.43)	0.015*
<3	1		1	
CXCL10, pg/mL				
≥690	0.34 (0.12–0.95)	0.040*	0.23 (0.07–0.69)	0.009*
<690	1		1	

*Note*: Hazard ratios were calculated using the Cox proportional hazard method.

Abbreviations: AFP, α‐fetoprotein; CI, confidence interval; CXCL10, C‐X‐C motif chemokine ligand 10; HR, hazard ratio; NLR, neutrophil‐to‐lymphocyte ratio; OS, overall survival.

* *p* < 0.05.

** *p* < 0.005.

## 4 DISCUSSION

As previously mentioned, serum CCL5 and CXCL10 are associated with hot immune features, and patients with advanced HCC and higher serum CCL5 and CXCL10 levels are more likely to benefit from ICI therapy.^15^ This study revealed the utility of serum CXCL10 levels after the introduction of Atez/Bev therapy for predicting prognosis among patients with BCLC stage C HCC.

CXCL10 is secreted by multiple cell types including monocytes, endothelial cells, fibroblasts, inflammatory cells, and tumor cells in response to interferon‐γ,^25^ and increased CXCL10 expression in tumor cells is important for anti‐tumor T cell responses.^26^ The CXCR3 chemokine system, which is associated with CXCL10, plays an important role in CD8+ T cell recruitment to tumors.^27^, ^28^ In a mouse study, CD8+ T cell infiltration in HCC was induced by CXCL10 expression,^29^ and patients with melanoma who responded to therapy had higher plasma CXCL9 and CXCL10 levels after ICI therapy.^16^ Albeit without significance, serum CXCL10 levels were numerically higher at baseline and at the start of the second course and more strongly increased from baseline to the start of the second course among responders than among non‐responders in the initial therapeutic response assessment of patients with BCLC stage C HCC (Figure 1). Based on these results, it is possible that the induction of CXCL10 promoted the infiltration of CD8+ T cells into the tumor and resulted in good therapeutic efficacy.

Our study identified increased serum CXCL10 levels during Atez/Bev therapy as a predictor of therapeutic efficacy in patients with BCLC stage C HCC, but these findings were not replicated in patients with BCLC stage A or B HCC. A previous study of the immune microenvironment in HCC revealed that the immune‐high subtype, which is characterized by increased B−/plasma‐cell and T‐cell infiltration, was associated with poorly differentiated HCC and PD‐L1 expression in both tumor and immune cells.^30^ It has been reported that inflamed (immune‐hot) HCC has a better response to ICI therapy.^31^ It is assumed that BCLC stage C HCC featuring distant metastasis and/or vascular invasion carries a higher risk of high‐grade malignancy. From these findings, we speculate that the induction of CXCL10 by ICI therapy differed in BCLC stage C patients, which might have influenced treatment efficacy.

In this study, serum CXCL10‐2c levels were independently associated with PFS and OS in patients with BCLC stage C HCC (Table 4 and Table S5). In addition, the Child–Pugh score at baseline and NLR at the start of the second course of therapy were independently associated with OS in this subgroup of patients. Liver function has been linked to the efficacy of Atez/Bev therapy.^32^ NLR at baseline and at the start of the second course is reported to be associated with the efficacy of Atez/Bev therapy, as NLR reflects the balance between the tumor‐promoting environment and anti‐tumor immune status.^33^, ^34^, ^35^ This is the first study, to the best of our knowledge, to reveal an association between serum CXCL10 levels during Atez/Bev therapy and therapeutic efficacy in patients with unresectable HCC.

Contrarily, serum CCL5 levels were not predictive of the efficacy of Atez/Bev. CCR5, the receptor for the chemokine CCL5, has tumor‐suppressing and tumor‐promoting roles.^36^, ^37^ Regarding tumor progression, the CCR5/CCL5 interaction is involved in the activation of the Akt pathway, which is related to the development of HCC.^38^ Conversely, in terms of anti‐tumor activity, CCR5 expression on CD8+ T cells is necessary for CD8+ T cell activation and migration to tumor sites.^36^ CCL5 itself attracts conventional type 1 dendritic cells to the tumor and promotes T cell infiltration into the tumor.^39^ Interestingly, our study revealed a correlation between baseline serum CCL5 levels and the neutrophil count (Table S3). Neutrophils are involved in the production of ligands that induce tumor cell proliferation and invasion and cytokines that induce angiogenesis.^40^ Meanwhile, serum CCL5‐2c levels were correlated with the lymphocyte count (Table S3). Lymphocytes are responsible for the immune function of the host, and decreased lymphocyte counts can impair hosts’ anti‐tumor immunity and worsen their prognosis.^41^ Thus, serum CCL5 levels were correlated with both tumor‐promoting markers at baseline and anti‐tumor markers after the introduction of Atez/Bev therapy. Therefore, we presume that these functions could explain the lack of an association between serum CCL5 levels and treatment efficacy in this study.

Our study had several important limitations. First, as a multicenter, retrospective, observational study, the possibility of selection bias cannot be excluded. Second, the cohort was not large. Additional studies in larger patient populations are needed to confirm the association between serum CXCL10 levels and Atez/Bev efficacy. Third, this study had an insufficient median follow‐up period (13.4 months). Fourth, we analyzed patients treated with Atez/Bev without stratification by treatment line. Finally, the efficacy of treatment regimens after Atez/Bev therapy, which can affect OS, was not examined.

In conclusion, this study demonstrated the utility of serum CXCL10 levels after the start of treatment predicting the efficacy of Atez/Bev therapy and patient prognosis in HCC. Future well‐designed prospective studies with large numbers of patients are desirable.

## AUTHOR CONTRIBUTIONS

**Takanori Suzuki:** Data curation (equal); writing – original draft (lead). **Kentaro Matsuura:** Writing – original draft (equal). **Yuta Suzuki:** Data curation (equal). **Fumihiro Okumura:** Data curation (equal). **Yoshihito Nagura:** Data curation (equal). **Satoshi Sobue:** Data curation (equal). **Sho Matoya:** Data curation (equal). **Tomokatsu Miyaki:** Data curation (equal). **Yoshihide Kimura:** Data curation (equal). **Atsunori Kusakabe:** Data curation (equal). **Satoshi Narahara:** Data curation (equal). **Takayuki Tokunaga:** Data curation (equal). **Katsuya Nagaoka:** Data curation (equal). **Keita Kuroyanagi:** Data curation (equal). **Hayato Kawamura:** Data curation (equal). **Kayoko Kuno:** Data curation (equal). **Kei Fujiwara:** Data curation (equal). **Shunsuke Nojiri:** Data curation (equal). **Hiromi Kataoka:** Supervision (equal). **Yasuhito Tanaka:** Supervision (equal).

## FUNDING INFORMATION

This research was supported by the Japan Society for the Promotion of Science (JSPS) KAKENHI (Grant Number: JP23K07359 to Kentaro Matsuura).

## CONFLICT OF INTEREST STATEMENT

Yasuhito Tanaka: Research funding from Janssen Pharmaceutical K.K., Gilead Sciences, AbbVie GK, GlaxoSmithKline PLC, Fujirebio Incorporation, and Sysmex Corporation and speaker's fees from AbbVie GK, Gilead Sciences, Chugai Pharmaceutical Co., Ltd., ASKA Pharmaceutical Holdings Co., Ltd., OTSUKA Pharmaceutical Co., Ltd., Takeda Pharmaceutical Co., Ltd., and GlaxoSmithKline PLC. Hiromi Kataoka: Honoraria from Takeda Pharmaceutical Co. and Otsuka Pharmaceutical Co. and fees for promotional materials from Eisai Co. and Otsuka Pharmaceutical Co.

## ETHICAL STATEMENT

*Approval of the research protocol*: The study protocol was approved by the Institutional Review Boards of Nagoya City University (approval number: 60‐21‐0065) and Kumamoto University Hospital (approval number: 2265) and implemented according to the Declaration of Helsinki. *Informed consent*: Written informed consent was obtained from all patients. *Registry and registration Nos*.: 60‐21‐0065 and 2265. *Animal studies*: N/A. *Research involving recombinant DNA*: N/A.

## Supporting information


Figures S1–S12.
Tables S1–S5.

## Data Availability

The authors confirm that the data supporting the findings of this study are available within the article and its supplementary materials.

## References

[1] Sung H , Ferlay J , Siegel RL , et al. Global cancer statistics 2020: GLOBOCAN estimates of incidence and mortality worldwide for 36 cancers in 185 countries. CA Cancer J Clin. 2021;71:209‐249. 33538338 10.3322/caac.21660

[2] Park JW , Chen M , Colombo M , et al. Global patterns of hepatocellular carcinoma management from diagnosis to death: the BRIDGE study. Liver Int. 2015;35:2155‐2166. 25752327 10.1111/liv.12818 PMC4691343

[3] Akinyemiju T , Abera S , Ahmed M , et al. The burden of primary liver cancer and underlying etiologies from 1990 to 2015 at the global, regional, and national level: results from the global burden of disease study 2015. JAMA Oncol. 2017;3:1683‐1691. 28983565 10.1001/jamaoncol.2017.3055 PMC5824275

[4] Llovet JM , Ricci S , Mazzaferro V , et al. Sorafenib in advanced hepatocellular carcinoma. N Engl J Med. 2008;359:378‐390. 18650514 10.1056/NEJMoa0708857

[5] Kudo M , Finn RS , Qin S , et al. Lenvatinib versus sorafenib in first‐line treatment of patients with unresectable hepatocellular carcinoma: a randomised phase 3 non‐inferiority trial. Lancet. 2018;391:1163‐1173. 29433850 10.1016/S0140-6736(18)30207-1

[6] Bruix J , Qin S , Merle P , et al. Regorafenib for patients with hepatocellular carcinoma who progressed on sorafenib treatment (RESORCE): a randomised, double‐blind, placebo‐controlled, phase 3 trial. Lancet. 2017;389:56‐66. 27932229 10.1016/S0140-6736(16)32453-9

[7] Zhu AX , Finn RS , Galle PR , Llovet JM , Kudo M . Ramucirumab in advanced hepatocellular carcinoma in REACH‐2: the true value of α‐fetoprotein. Lancet Oncol. 2019;20:e191. 30942178 10.1016/S1470-2045(19)30165-2

[8] Abou‐Alfa GK , Meyer T , Cheng AL , et al. Cabozantinib in patients with advanced and progressing hepatocellular carcinoma. N Engl J Med. 2018;379:54‐63. 29972759 10.1056/NEJMoa1717002 PMC7523244

[9] Finn RS , Qin S , Ikeda M , et al. Atezolizumab plus bevacizumab in unresectable hepatocellular carcinoma. N Engl J Med. 2020;382:1894‐1905. 32402160 10.1056/NEJMoa1915745

[10] Cheng AL , Qin S , Ikeda M , et al. Updated efficacy and safety data from IMbrave150: atezolizumab plus bevacizumab vs. sorafenib for unresectable hepatocellular carcinoma. J Hepatol. 2022;76:862‐873. 34902530 10.1016/j.jhep.2021.11.030

[11] Adams S , Schmid P , Rugo HS , et al. Pembrolizumab monotherapy for previously treated metastatic triple‐negative breast cancer: cohort a of the phase II KEYNOTE‐086 study. Ann Oncol. 2019;30:397‐404. 30475950 10.1093/annonc/mdy517

[12] Antonarakis ES , Piulats JM , Gross‐Goupil M , et al. Pembrolizumab for treatment‐refractory metastatic castration‐resistant prostate cancer: multicohort, open‐label phase II KEYNOTE‐199 study. J Clin Oncol. 2020;38:395‐405. 31774688 10.1200/JCO.19.01638 PMC7186583

[13] Finn RS , Ryoo BY , Merle P , et al. Pembrolizumab as second‐line therapy in patients with advanced hepatocellular carcinoma in KEYNOTE‐240: a randomized, double‐blind, phase III trial. J Clin Oncol. 2020;38:193‐202. 31790344 10.1200/JCO.19.01307

[14] Bruni D , Angell HK , Galon J . The immune contexture and Immunoscore in cancer prognosis and therapeutic efficacy. Nat Rev Cancer. 2020;20:662‐680. 32753728 10.1038/s41568-020-0285-7

[15] Montironi C , Castet F , Haber PK , et al. Inflamed and non‐inflamed classes of HCC: a revised immunogenomic classification. Gut. 2023;72:129‐140. 35197323 10.1136/gutjnl-2021-325918 PMC9395551

[16] Chow MT , Ozga AJ , Servis RL , et al. Intratumoral activity of the CXCR3 chemokine system is required for the efficacy of anti‐PD‐1 therapy. Immunity. 2019;50:1498‐1512.e5. 31097342 10.1016/j.immuni.2019.04.010 PMC6527362

[17] Hosoda S , Suda G , Sho T , et al. Low baseline CXCL9 predicts early progressive disease in unresectable HCC with atezolizumab plus bevacizumab treatment. Liver Cancer. 2022;12:156‐170. 37325489 10.1159/000527759 PMC10267515

[18] Myojin Y , Kodama T , Sakamori R , et al. Interleukin‐6 is a circulating prognostic biomarker for hepatocellular carcinoma patients treated with combined immunotherapy. Cancers (Basel). 2022;14:883. 35205631 10.3390/cancers14040883 PMC8870238

[19] Yang H , Kang B , Ha Y , et al. High serum IL‐6 correlates with reduced clinical benefit of atezolizumab and bevacizumab in unresectable hepatocellular carcinoma. JHEP Rep. 2023;5:100672. 36866388 10.1016/j.jhepr.2023.100672 PMC9972403

[20] Llovet JM , Brú C , Bruix J . Prognosis of hepatocellular carcinoma: the BCLC staging classification. Semin Liver Dis. 1999;19:329‐338. 10518312 10.1055/s-2007-1007122

[21] Johnson PJ , Berhane S , Kagebayashi C , et al. Assessment of liver function in patients with hepatocellular carcinoma: a new evidence‐based approach‐the ALBI grade. J Clin Oncol. 2015;33:550‐558. 25512453 10.1200/JCO.2014.57.9151 PMC4322258

[22] Eisenhauer EA , Therasse P , Bogaerts J , et al. New response evaluation criteria in solid tumours: revised RECIST guideline (version 1.1). Eur J Cancer. 2009;45:228‐247. 19097774 10.1016/j.ejca.2008.10.026

[23] Kanda Y . Investigation of the freely available easy‐to‐use software ‘EZR’ for medical statistics. Bone Marrow Transplant. 2013;48:452‐458. 23208313 10.1038/bmt.2012.244 PMC3590441

[24] Persano M , Rimini M , Tada T , et al. Role of the prognostic nutritional index in predicting survival in advanced hepatocellular carcinoma treated with atezolizumab plus bevacizumab. Oncology. 2023;101:283‐291. 36657420 10.1159/000528818

[25] Tokunaga R , Zhang W , Naseem M , et al. CXCL9, CXCL10, CXCL11/CXCR3 axis for immune activation‐a target for novel cancer therapy. Cancer Treat Rev. 2018;63:40‐47. 29207310 10.1016/j.ctrv.2017.11.007 PMC5801162

[26] Sistigu A , Yamazaki T , Vacchelli E , et al. Cancer cell‐autonomous contribution of type I interferon signaling to the efficacy of chemotherapy. Nat Med. 2014;20:1301‐1309. 25344738 10.1038/nm.3708

[27] Mikucki ME , Fisher DT , Matsuzaki J , et al. Non‐redundant requirement for CXCR3 signalling during tumoricidal T‐cell trafficking across tumour vascular checkpoints. Nat Commun. 2015;6:7458. 26109379 10.1038/ncomms8458 PMC4605273

[28] Elia G , Fallahi P . Hepatocellular carcinoma and CXCR3 chemokines: a narrative review. Clin Ter. 2017;168:e37‐e41. 28240761 10.7417/CT.2017.1980

[29] Shigeta K , Matsui A , Kikuchi H , et al. Regorafenib combined with PD1 blockade increases CD8 T‐cell infiltration by inducing CXCL10 expression in hepatocellular carcinoma. J Immunother Cancer. 2020;8:e001435. 33234602 10.1136/jitc-2020-001435 PMC7689089

[30] Kurebayashi Y , Ojima H , Tsujikawa H , et al. Landscape of immune microenvironment in hepatocellular carcinoma and its additional impact on histological and molecular classification. Hepatology. 2018;68:1025‐1041. 29603348 10.1002/hep.29904

[31] Donne R , Lujambio A . The liver cancer immune microenvironment: therapeutic implications for hepatocellular carcinoma. Hepatology. 2023;77:1773‐1796. 35989535 10.1002/hep.32740 PMC9941399

[32] Tanaka T , Hiraoka A , Tada T , et al. Therapeutic efficacy of atezolizumab plus bevacizumab treatment for unresectable hepatocellular carcinoma in patients with child‐Pugh class a or B liver function in real‐world clinical practice. Hepatol Res. 2022;52:773‐783. 35633504 10.1111/hepr.13797

[33] Ochi H , Kurosaki M , Joko K , et al. Usefulness of neutrophil‐to‐lymphocyte ratio in predicting progression and survival outcomes after atezolizumab‐bevacizumab treatment for hepatocellular carcinoma. Hepatol Res. 2022;53:61‐71. 36070216 10.1111/hepr.13836

[34] Tada T , Kumada T , Hiraoka A , et al. Neutrophil‐lymphocyte ratio predicts early outcomes in patients with unresectable hepatocellular carcinoma treated with atezolizumab plus bevacizumab: a multicenter analysis. Eur J Gastroenterol Hepatol. 2022;34:698‐706. 35170529 10.1097/MEG.0000000000002356

[35] Matoya S , Suzuki T , Matsuura K , et al. The neutrophil‐to‐lymphocyte ratio at the start of the second course during atezolizumab plus bevacizumab therapy predicts therapeutic efficacy in patients with advanced hepatocellular carcinoma: a multicenter analysis. Hepatol Res. 2023;53:511‐521. 36723964 10.1111/hepr.13886

[36] González‐Martín A , Mira E , Mañes S . CCR5 in cancer immunotherapy: more than an "attractive" receptor for T cells. Onco Targets Ther. 2012;1:106‐108. 10.4161/onci.1.1.17995 PMC3376953 22720226

[37] Velasco‐Velázquez M , Xolalpa W , Pestell RG . The potential to target CCL5/CCR5 in breast cancer. Expert Opin Ther Targets. 2014;18:1265‐1275. 25256399 10.1517/14728222.2014.949238

[38] Singh SK , Mishra MK , Rivers BM , Gordetsky JB , Bae S , Singh R . Biological and clinical significance of the CCR5/CCL5 axis in hepatocellular carcinoma. Cancers (Basel). 2020;12:883. 32260550 10.3390/cancers12040883 PMC7226629

[39] Berraondo P , Ochoa MC , Olivera I , Melero I . Immune desertic landscapes in hepatocellular carcinoma shaped by beta‐catenin activation. Cancer Discov. 2019;9:1003‐1005. 31371324 10.1158/2159-8290.CD-19-0696

[40] Terzić J , Grivennikov S , Karin E , Karin M . Inflammation and colon cancer. Gastroenterology. 2010;138:2101‐2114.e5. 20420949 10.1053/j.gastro.2010.01.058

[41] Lin EY , Pollard JW . Role of infiltrated leucocytes in tumour growth and spread. Br J Cancer. 2004;90:2053‐2058. 15164120 10.1038/sj.bjc.6601705 PMC2410285

