# Supplementary material for: Serum CXCL10 levels at the start of the second course of atezolizumab plus bevacizumab therapy predict therapeutic efficacy in patients with advanced BCLC stage C hepatocellular carcinoma: A multicenter analysis
Source: Cancer Med. 2023 Dec 22;13(1):e6876. doi: 10.1002/cam4.6876 (PMC10807580; doi:10.1002/cam4.6876)
Supplement: Supplementary file 1 — Figures S1–S12. Tables S1–S5. [file CAM4-13-e6876-s001.docx]

**Supplementary Information**

**Title:** Serum CXCL10 levels at the start of the second course of atezolizumab plus bevacizumab therapy predict therapeutic efficacy in patients with advanced BCLC stage C hepatocellular carcinoma: A multicenter analysis

**Author:** Takanori Suzuki, Kentaro Matsuura, Yuta Suzuki, Fumihiro Okumura, Yoshihito Nagura, Satoshi Sobue, Sho Matoya, Tomokatsu Miyaki, Yoshihide Kimura, Atsunori Kusakabe, Satoshi Narahara, Takayuki Tokunaga, Katsuya Nagaoka, Keita Kuroyanagi, Hayato Kawamura, Kayoko Kuno, Kei Fujiwara, Shunsuke Nojiri, Hiromi Kataoka, Yasuhito Tanaka

Supplementary Figure1………………………………………………….………….P2

Supplementary Figure2…………………………………………………….……... P3

Supplementary Figure3…………………………………………………….……... P4

Supplementary Figure4…………………………………………………….……... P5

Supplementary Figure5…………………………………………………….……... P6

Supplementary Figure6…………………………………………………….……... P7

Supplementary Figure7…………………………………………………….……... P8

Supplementary Figure8…………………………………………………….……... P9

Supplementary Figure9……………………………………………………... P10

Supplementary Figure10…………………………………………………….…... P11

Supplementary Figure11………………………………………………………... P12

Supplementary Figure12………………………………………………………... P13

Supplementary Table1………………………………………………….……. P14-16

Supplementary Table2…………………………………………………... P17,18

Supplementary Table3…………………………………………………….……... P19

Supplementary Table4…………………………………………………….……... P20

Supplementary Table5……………………………………………….…. P21,22

**Supplementary Figure 1. The flowchart of the regimens before and after Atez/Bev therapy.**

**
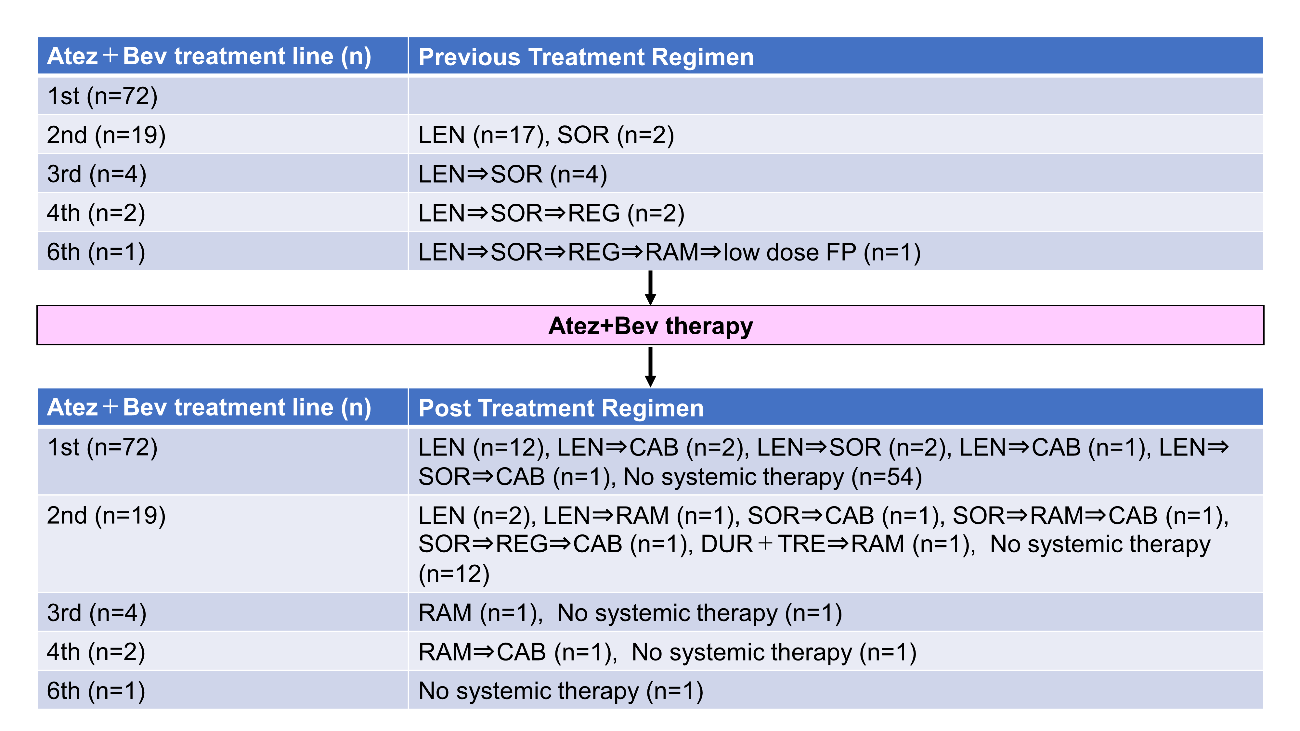
**

**
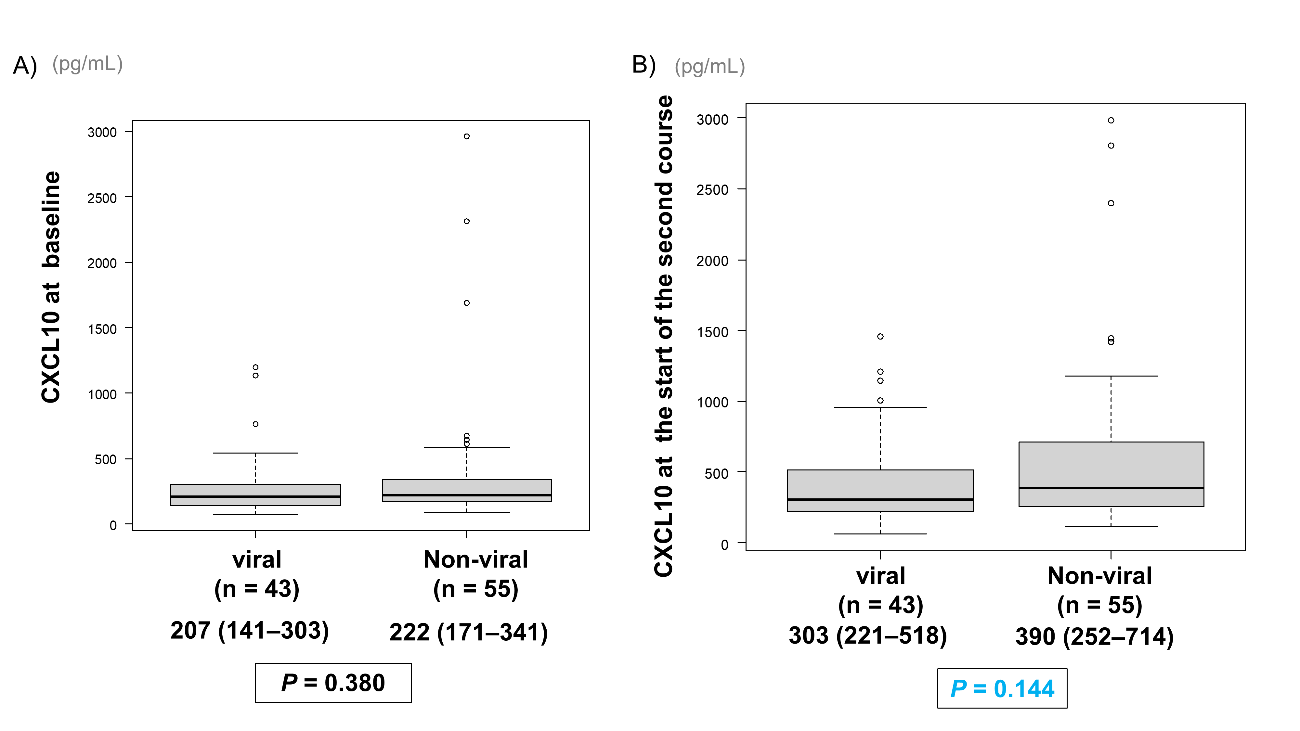
Supplementary Figure 2. Comparison of serum CXCL10 levels at baseline and the start of the second course stratified by etiology.**

**
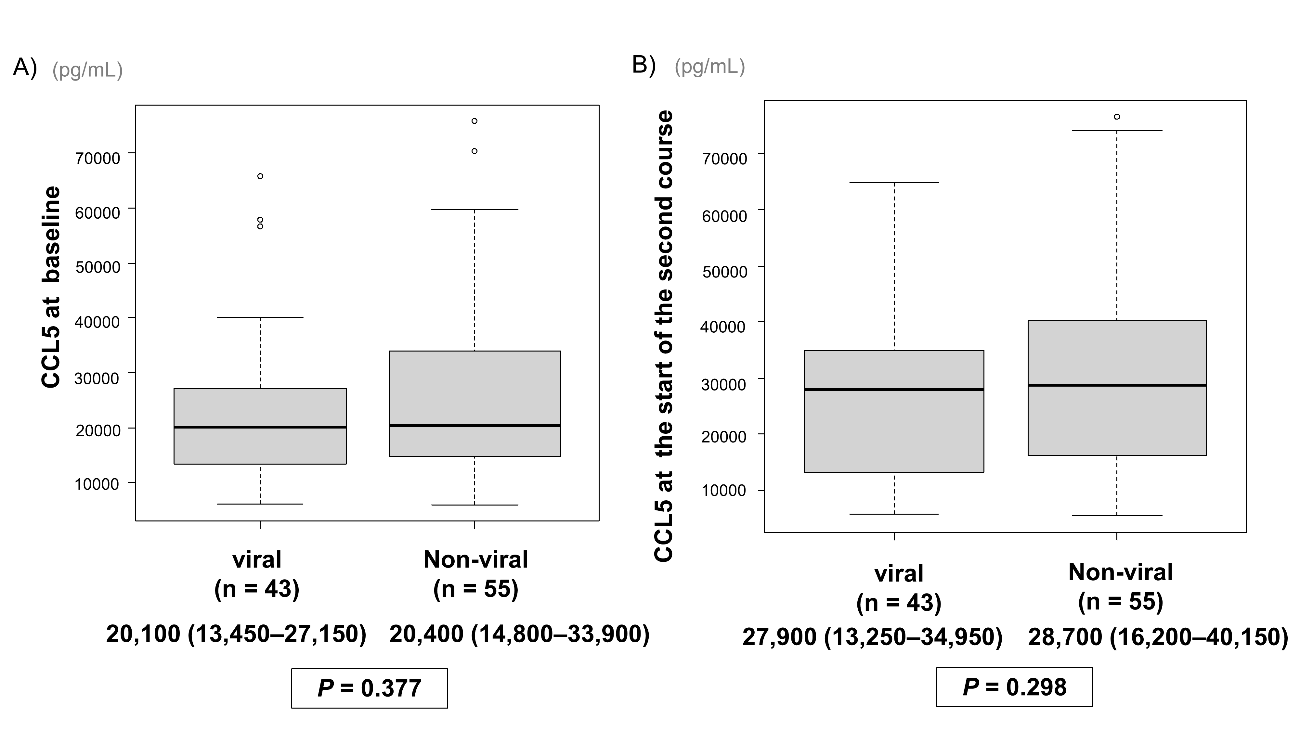
Supplementary Figure 3. Comparison of serum CCL5 levels at baseline and the start of the second course stratified by etiology.**

**Supplementary Figure 4. Comparison of serum CXCL10 levels at baseline and the start of the second course stratified by treatment line.**

**
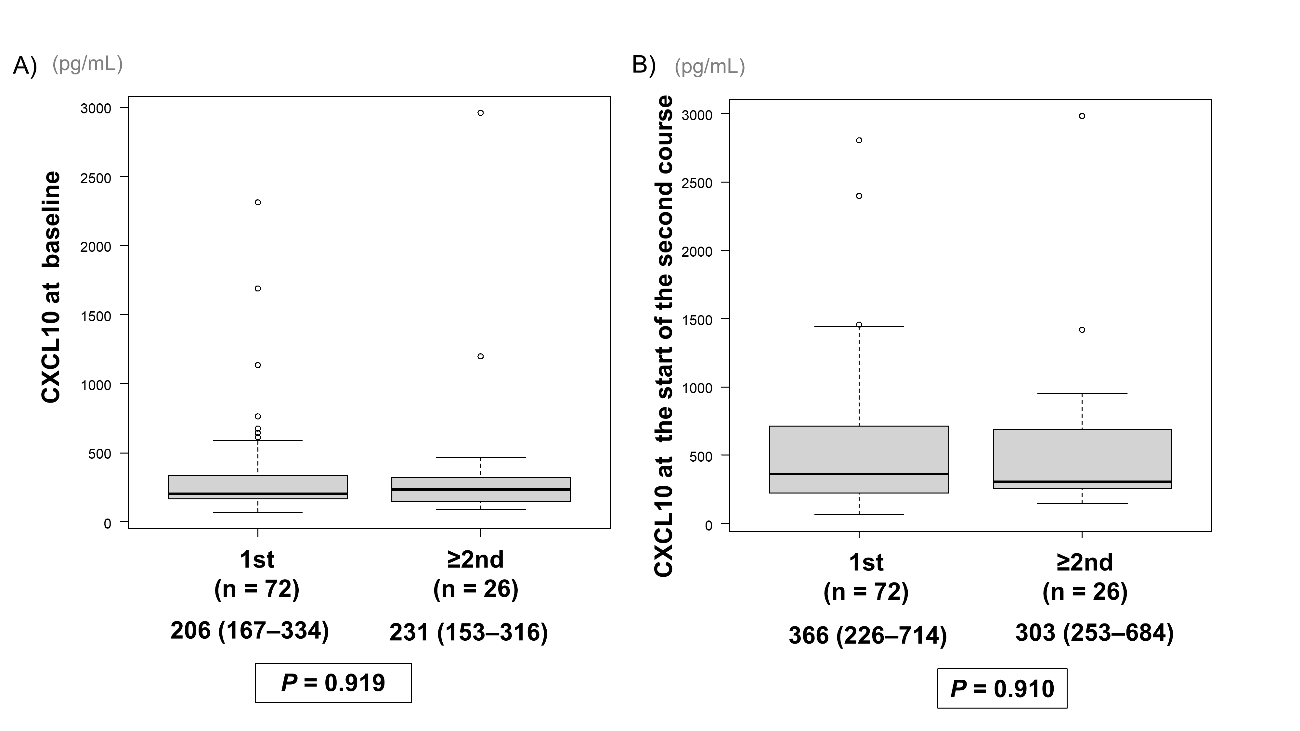
**

**
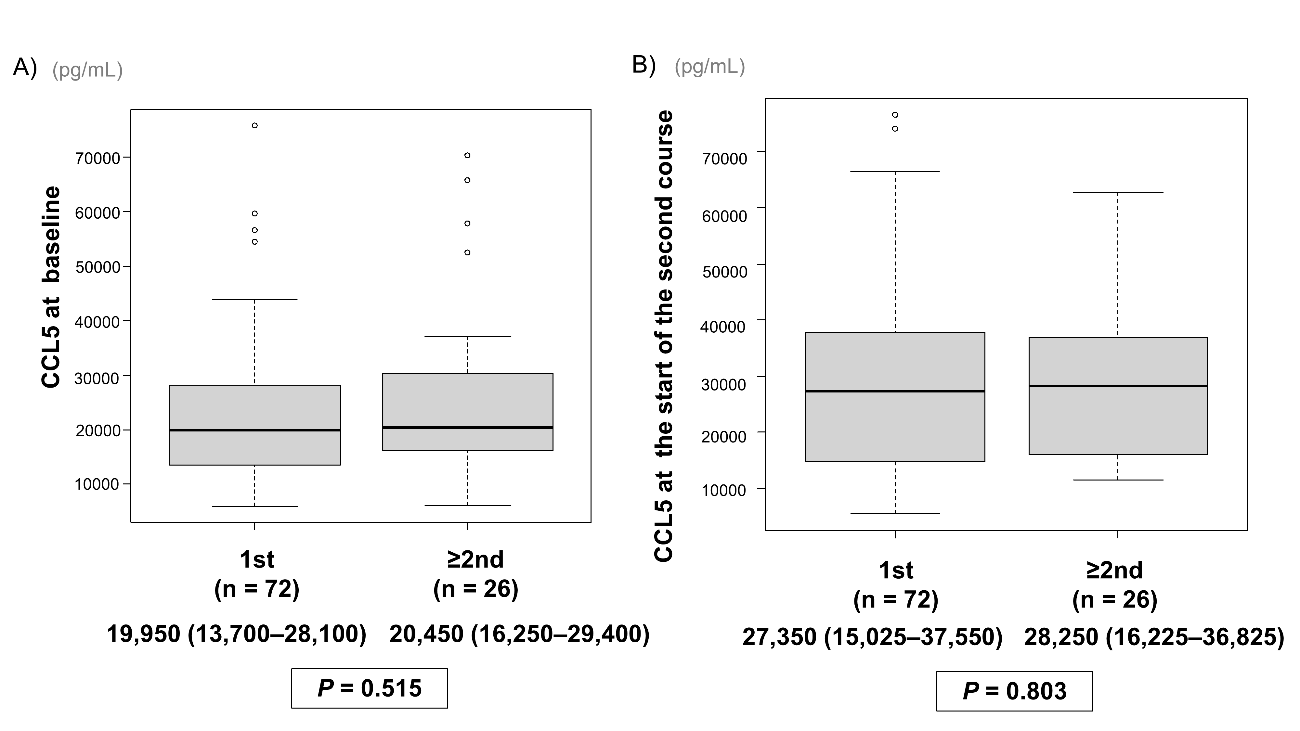
Supplementary Figure 5. Comparison of serum CCL5 levels at baseline and the start of the second course stratified by treatment line.**

**Supplementary Figure 6. Distribution of the initial therapeutic responses, OS, and PFS in all 98 study patients treated with Atez/Bev therapy.**


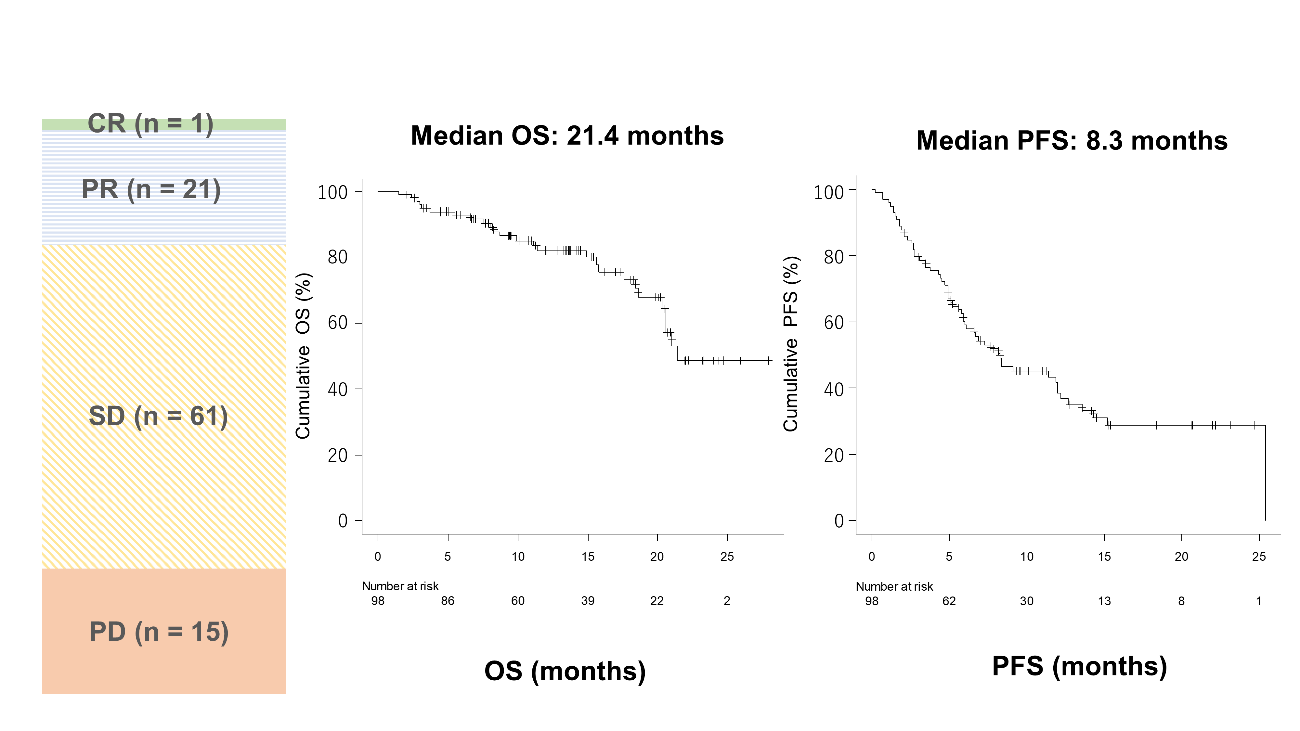


**Supplementary Figure 7. Comparison of serum CXCL10 levels at baseline and the start of the second course stratified by BCLC stage.**


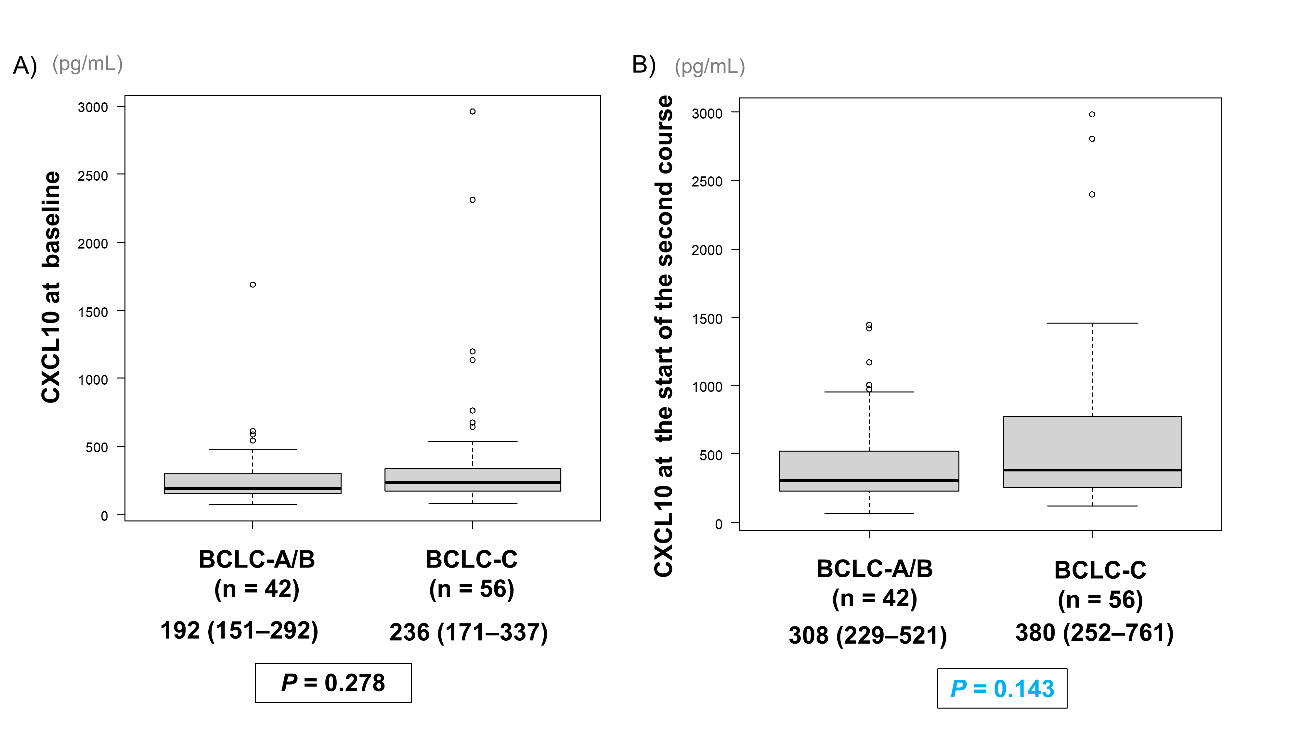


**Supplementary Figure 8. Comparison of serum CCL5 levels at baseline and the start of the second course stratified by BCLC stage.**


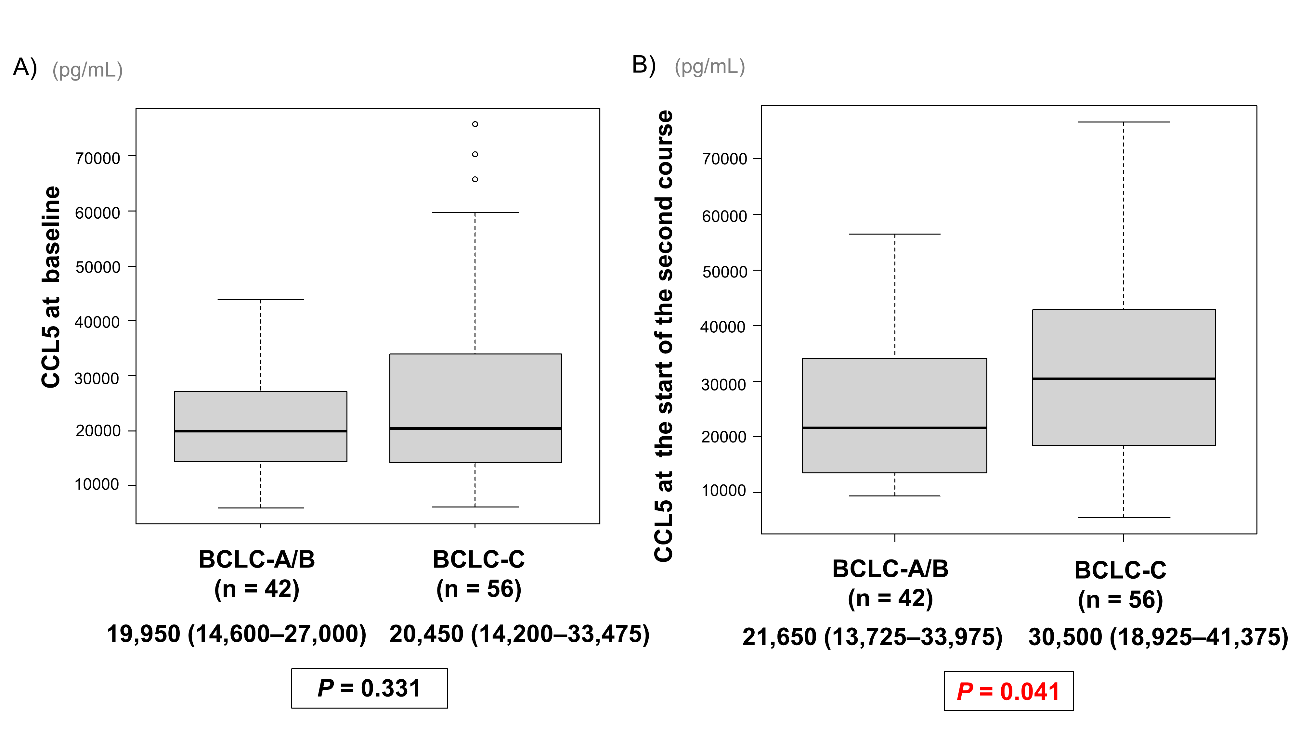


**Supplementary Figure 9.** **Changes of serum CCL5 levels from baseline to the start of the second course stratified by the initial therapeutic response in patients with BCLC stage C HCC.**


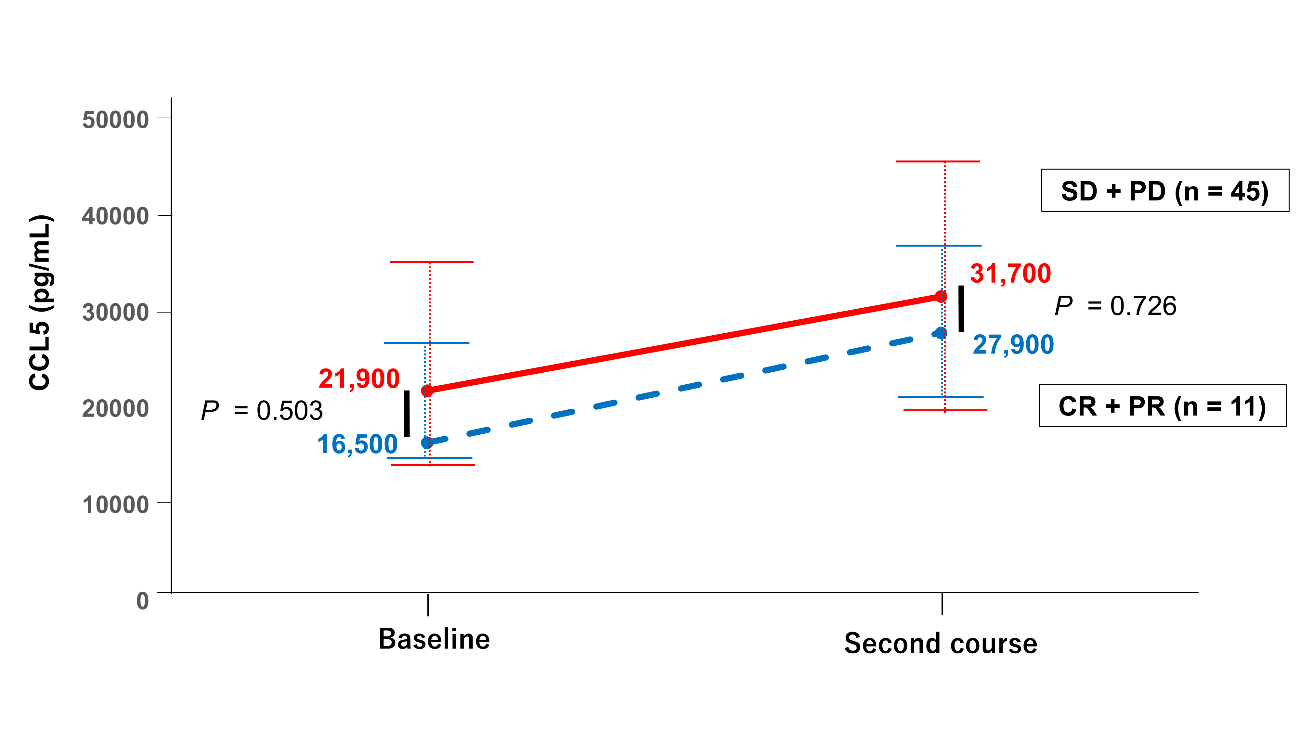


**Supplementary Figure 10. ROC curve analyses for discriminating therapeutic responses using serum CXCL10 levels and at baseline and the start of the second course.**


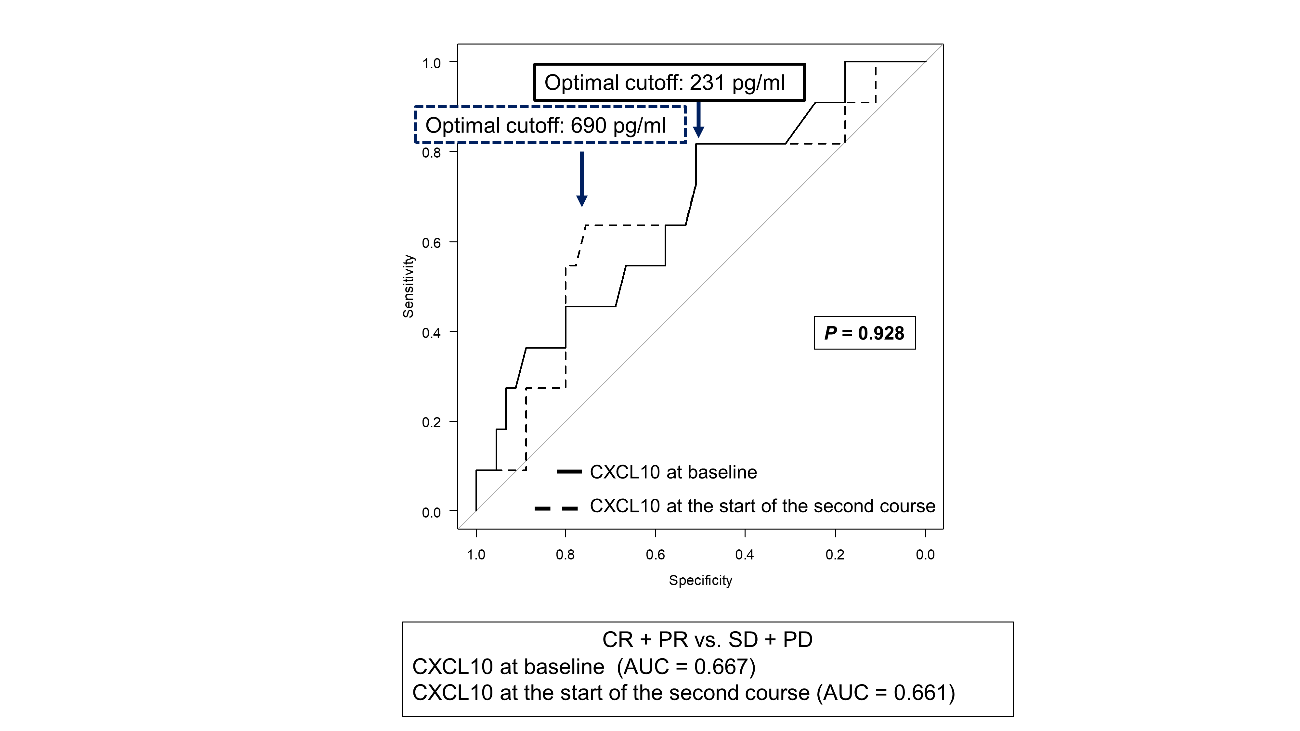


**Supplementary Figure 11. OS and PFS stratified by serum CXCL10 levels at the start of Atez/Bev therapy.**


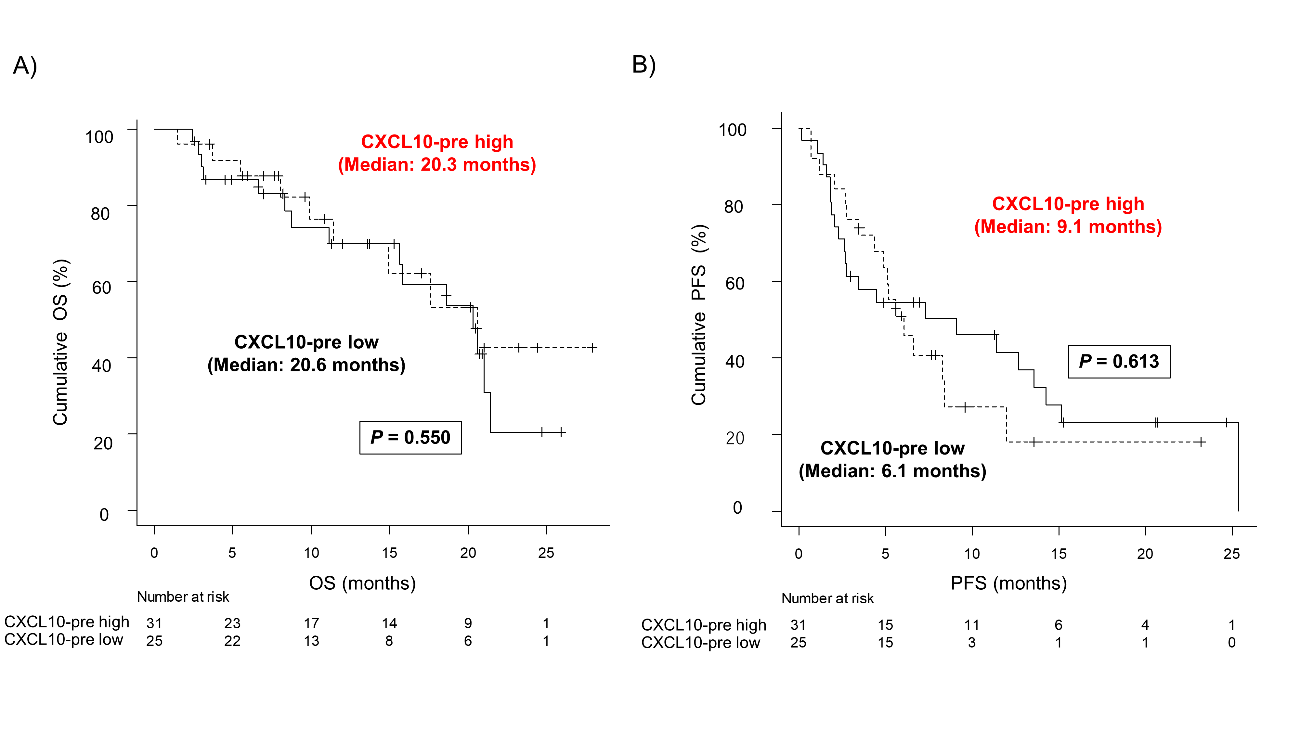


**Supplementary Figure 12. OS and PFS stratified by serum CXCL10 levels at the start of the second course of Atez/Bev therapy in patients with distant metastases.**


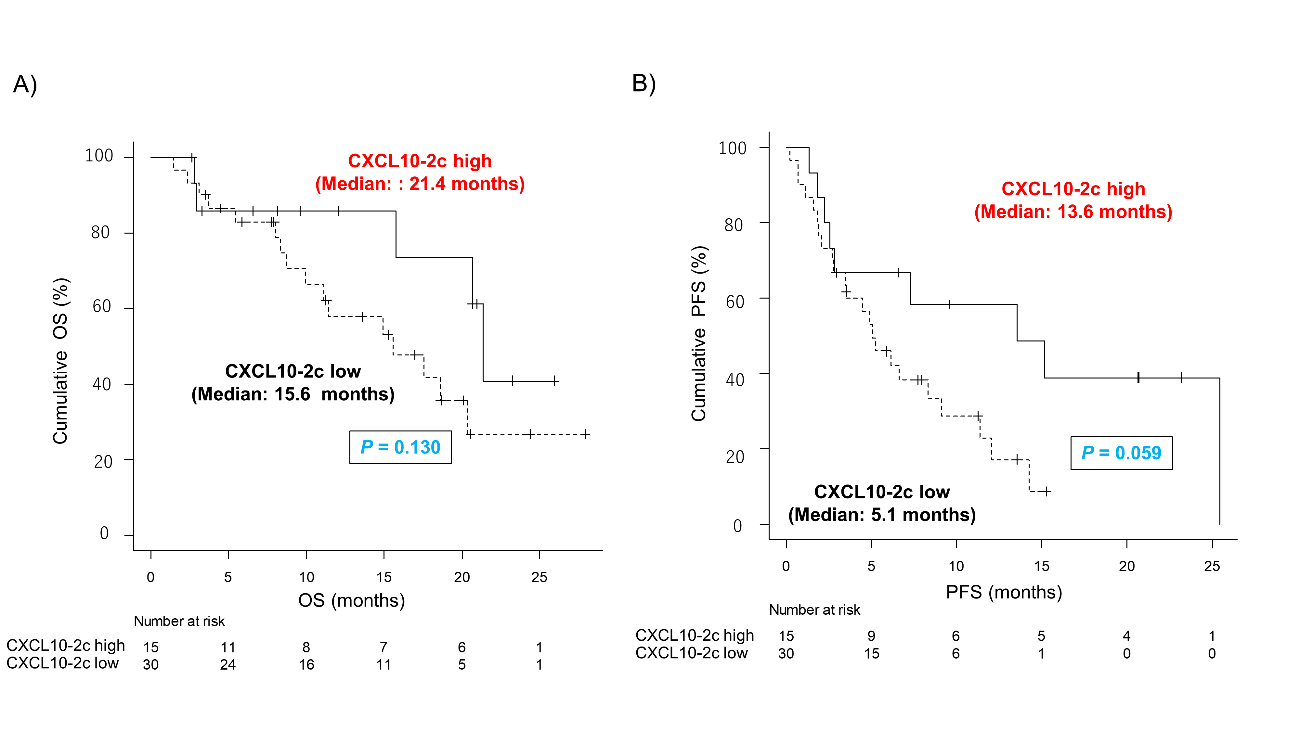


**Supplementary Table 1. Comparison of patient characteristics between responders (CR + PR) and non-responders (SD + PD) in the initial evaluation**

| **Characteristics** | **CR + PR (n = 22)** | **SD + PD (n = 76)** | ***P*-value** |
| --- | --- | --- | --- |
| **Baseline** |  |  |  |
| **Age, years** | **75 (66–79)** | **73 (66–78)** | **0.433** |
| **Gender, male/female** | **17/5** | **61/15** | **0.768** |
| **Etiology, viral/non-viral** | **7/15** | **36/40** | **0.229** |
| **Line of systemic chemotherapy, 1st/≥2nd** | **18/4** | **54/22** | **0.416** |
| **BMI, kg/m^2^** | **24.4 (21.9–27.1)** | **23.2 (20.7–26.5)** | **0.362** |
| **WBCs, /μL** | **5100 (4725–6225)** | **4950 (3700–6325)** | **0.548** |
| **Neutrophils, /μL** | **3523 (2541–4041)** | **3025 (2066–4000)** | **0.385** |
| **Lymphocytes, /μL** | **1100 (837–1,464)** | **1259 (899–1560)** | **0.490** |
| **NLR** | **2.95 (2.03–3.89)** | **2.48 (1.56–3.80)** | **0.338** |
| **PT, %** | **93.6 (88.8–104.4)** | **99.0 (87.0–107.0)** | **0.695** |
| **AST, U/L** | **43 (33–59)** | **38 (27–50)** | **0.220** |
| **ALT, U/L** | **31 (18–45)** | **27 (20–37)** | **0.443** |
| **Alb, g/dL** | **3.7 (3.4–3.9)** | **3.7 (3.4–4.0)** | **1.000** |
| **T.Bil, mg/dL** | **0.8 (0.6–1.0)** | **0.7 (0.5–1.2)** | **0.481** |
| **AFP, ng/mL** | **20.5 (9.0–1003.6)** | **15.7 (4.7–253.3)** | **0.367** |
| **PIVKA-II, mAU/mL** | **218 (44–532)** | **270 (52–1772)** | **0.595** |
| **ALBI score** | **−2.31 (−2.69 to −2.09)** | **−2.36 (−2.63 to −2.11)** | **0.905** |
| **mALBI grade, 1/2a/2b/3** | **7(32%)/6(27%)/9(41%)/0(0%)** | **22(29%)/25(33%)/27(36%)/2(3%)** | **0.920** |
| **Child–Pugh score: 5/6/7/8** | **12(55%)/9(41%)/1(5%)/0(0%)** | **37(49%)/26(34%)/11(14%)/2(3%)** | **0.635** |
| **BCLC stage, A/B/C** | **2(9%)/9(41%)/11(50%)** | **4(5%)/27(36%)/45(59%)** | **0.625** |
| **Extrahepatic metastasis, yes/no** | **8(36%)/14(64%)** | **37(49%)/39(51%)** | **0.341** |
| **CXCL10, pg/mL** | **243.0 (164.3–519.8)** | **196.5 (164.3–315.0)** | **0.150** |
| **CCL5, pg/mL** | **20,700 (14,825–33,100)** | **20,250 (13,800–27,800)** | **0.825** |
|  |  |  |  |
| **Start of the second course** |  |  |  |
| **Neutrophils, /μL** | **2924 (2183–3589)** | **2813 (1958–4194)** | **0.867** |
| **Lymphocyte, /μL** | **1579 (1118–1935)** | **1109 (800–1651)** | **0.057** |
| **NLR** | **2.02 (1.16–2.92)** | **2.57 (1.60–4.18)** | **0.170** |
| **CXCL10, pg/mL** | **373.5 (243.0–840.0)** | **345.0 (231.0–666.8)** | **0.342** |
| **CCL5, pg/mL** | **29,150 (18,825–40,700)** | **27,350 (14,950–36,600)** | **0.459** |

Data from all patients are expressed as numbers for categorical data and medians (first–third quartiles) for non-categorical data. Categorical variables were compared between the groups using Fisher’s exact test, and non-categorical variables were compared using the Mann–Whitney *U* test. *, *P* < 0.05; **, *P* < 0.005. Abbreviations: CR, complete response; PR, partial response; SD, stable disease; PD, progressive disease; BMI, body mass index; WBCs, white blood cells; NLR, neutrophil-to-lymphocyte ratio; PT, prothrombin time; AST, aspartate transaminase; ALT, alanine transaminase; Alb, albumin; T.Bil, total bilirubin; AFP, α-fetoprotein; PIVKA-II, protein induced by vitamin K absence or antagonist-II; ALBI score, albumin–bilirubin score; mALBI grade, modified albumin–bilirubin grade; BCLC, Barcelona Clinic Liver Cancer; CXCL10, C-X-C motif chemokine ligand 10; CCL5, C-C motif chemokine ligand 5.

**Supplementary Table 2. Clinical characteristics of patients with Barcelona Clinic Liver Cancer stage C hepatocellular carcinoma**

| **Baseline** | **n = 56** |
| --- | --- |
| **Age, years** | **72 (65–78)** |
| **Gender, male/female** | **45/11** |
| **BMI, kg/m^2^** | **23.2 (20.7–25.2)** |
| **ECOG PS, 0/1** | **49/7** |
| **Etiology, HBV/HCV/alcohol/others** | **8/16/10/22** |
| **Treatment line, 1^st/^2^nd/^3^rd/^4^th^/6^th^** | **37/12/4/2/1** |
| **WBCs, µL** | **5350 (4175–6825)** |
| **Neutrophils, µL** | **3422 (2373–4348)** |
| **Lymphocytes, μL** | **1,261 (794–1609)** |
| **NLR** | **2.76 (1.62–4.45)** |
| **Hb, g/dL** | **12.7 (10.8–14.0)** |
| **PLT, × 10^4^/μL** | **15.9 (11.3–21.3)** |
| **PT, %** | **101.0 (88.0–107.5)** |
| **AST, U/L** | **40 (28–51)** |
| **ALT, U/L** | **27 (18–38)** |
| **Alb, g/dL** | **3.6 (3.3–3.9)** |
| **T.Bil, mg/dL** | **0.8 (0.7–1.1)** |
| **AFP, ng/mL** | **46.3 (5.2–2751.6)** |
| **PIVKA-II, mAU/mL** | **682.5 (58.3–5959.3)** |
| **ALBI score** | **−2.33 (−2.57 to −2.09)** |
| **mALBI grade, 1/2a/2b/3** | **14(25%)/16(29%)/25(45%)/1(2%)** |
| **Child–Pugh score, 5/6/7/8** | **26(46%)/20(36%)/9(16%)/1(2%)** |
| **MVI, yes/no** | **15(27%)/41(73%)** |
| **Extrahepatic metastasis, yes/no** | **45(80%)/11(20%)** |
| **CXCL10, pg/mL** | **235.5 (171.0–336.8)** |
| **CCL5, pg/mL** | **20,450 (14,200–33,475)** |
|  |  |
| **Start of the second course** |  |
| **Neutrophils, µL** | **3192 (2248–4419)** |
| **Lymphocytes, μL** | **1208 (726–1700)** |
| **NLR** | **2.57 (1.71–4.52)** |
| **CXCL10, pg/mL** | **379.5 (252.0–761.3)** |
| **CCL5, pg/mL** | **30,500 (18,925–41,375)** |

Data from all patients are expressed as numbers for categorical data and medians (first–third quartiles) for non-categorical data. Abbreviations: BMI, body mass index; ECOG PS, ECOG performance status; HBV, hepatitis B virus; HCV, hepatitis C virus; WBCs, white blood cells; NLR, neutrophil-to-lymphocyte ratio; PLT, platelet; PT, prothrombin time; AST, aspartate transaminase; ALT, alanine transaminase; Alb, albumin; T.Bil, total bilirubin; AFP, α-fetoprotein; PIVKA-II, protein induced by vitamin K absence or antagonist-II; ALBI score, albumin–bilirubin score; MVI, major vascular invasion; CXCL10, C-X-C motif chemokine ligand 10; CCL5, C-C motif chemokine ligand 5.

**Supplementary Table 3. Correlations of serum CCL5 levels with other clinical parameters**

|  | **CCL5 at baseline** | | **CCL5 at the start of the**  **second course** | |
| --- | --- | --- | --- | --- |
| **Factors** | **r** | ***P*-value** | **r** | ***P*-value** |
| **WBCs** | 0.368 | <0.001****** | 0.346 | <0.001****** |
| **Neutrophils** | 0.415 | <0.001****** | 0.310 | 0.002****** |
| **Lymphocytes** | 0.067 | 0.515 | 0.254 | 0.013****** |
| **NLR** | 0.317 | 0.001****** | 0.066 | 0.526 |
| **AFP** | 0.328 | 0.001****** |  |  |
| **PIVKA-II** | 0.500 | <0.001****** |  |  |
| **CXCL10** | 0.053 | 0.607 | 0.057 | 0.576 |

Correlation coefficients were calculated using Pearson’s correlation test. *, *P* < 0.05; **, *P* < 0.005. Abbreviations: CCL5, C-C motif chemokine ligand 5; WBC, white blood cells; NLR, neutrophil-to-lymphocyte ratio; AFP, α-fetoprotein; PIVKA-II, protein induced by vitamin K absence or antagonist-II; CXCL10, C-X-C motif chemokine ligand 10.

**Supplementary Table 4. Correlations of serum CXCL10 levels with other clinical parameters**

|  | **CXCL10 at baseline** | | **CXCL-10 at the start of the second course** | |
| --- | --- | --- | --- | --- |
| **Factors** | **r** | ***P*-value** | **r** | ***P*-value** |
| **WBCs** | −0.019 | 0.855 | 0.039 | 0.707 |
| **Neutrophils** | 0.017 | 0.865 | 0.019 | 0.858 |
| **Lymphocytes** | −0.203 | 0.045***** | −0.003 | 0.978 |
| **NLR** | 0.248 | 0.014***** | −0.003 | 0.981 |
| **AFP** | 0.087 | 0.394 |  |  |
| **PIVKA-II** | 0.102 | 0.322 |  |  |
| **CCL5** | 0.053 | 0.607 | 0.057 | 0.576 |

Correlation coefficients were calculated using Pearson’s correlation test. *, *P* < 0.05; **, *P* < 0.005. Abbreviations: CXCL10, C-X-C motif chemokine ligand 10; WBCs, white blood cells; NLR, neutrophil-to-lymphocyte ratio; AFP, α-fetoprotein; PIVKA-II, protein induced by vitamin K absence or antagonist-II; CCL5, C-C motif chemokine ligand 5.

**Supplementary Table 5. Factors associated with progression-free survival in patients with Barcelona Clinic Liver Cancer stage C hepatocellular carcinoma who received atezolizumab plus bevacizumab**

| **Factor** | **Univariate analysis**  **SD (n =62)** | | **Multivariate analysis** | |
| --- | --- | --- | --- | --- |
|  | **HR (95% CI)** | ***P*-value** | **HR (95% CI)** | ***P*-value** |
| **Baseline** |  |  |  |  |
| **Age, years**  **≥70**  **<70** | **0.66 (0.34–1.28)**  **1** | **0.220** |  |  |
| **Gender**  **Male**  **Female** | **0.56 (0.27–1.16)**  **1** | **0.119** |  |  |
| **Etiology**  **Non-viral**  **Viral** | **0.74 (0.39–1.42)**  **1** | **0.372** |  |  |
| **Line of systemic chemotherapy**  **≥2nd**  **1st** | **1.79 (0.95–3.41)**  **1** | **0.073** |  |  |
| **NLR**  **≥3**  **<3** | **1.51 (0.79–2.86)**  **1** | **0.211** |  |  |
| **AFP, ng/mL**  **≥400**  **<400** | **0.95 (0.49–1.85)**  **1** | **0.888** |  |  |
| **Child–Pugh score**  **≥6**  **5** | **1.49 (0.77–2.87)**  **1** | **0.232** |  |  |
| **CXCL10, pg/mL**  **≥231**  **<231** | **0.84 (0.44–1.63)**  **1** | **0.611** |  |  |
| **Start of the second course** |  |  |  |  |
| **NLR**  **≥3**  **<3** | **1.74 (0.92–3.30)**  **1** | **0.090** |  |  |
| **CXCL10, pg/mL**  **≥690**  **<690** | **0.39 (0.18–0.85)**  **1** | **0.017*** |  |  |

Hazard ratios were calculated by the Cox proportional hazard method. *, *P* < 0.05; **, *P* < 0.005. Abbreviations: NLR, neutrophil-to-lymphocyte ratio; AFP, α-fetoprotein; CXCL10, C-X-C motif chemokine ligand 10; HR, hazard ratio; CI, confidence interval.
